# Multi-Year Characterisation of Drought Adaptation and Grain Quality in Synthetic Hexaploid Wheat Genotypes

**DOI:** 10.3390/plants15193054

**Published:** 2026-10-07

**Authors:** Metanet Babayeva, Shamsiyya Mammadova, Aziza Huseynova, Aynura Pashayeva, Turana Isgandarova, Saida Zulfugarova, Mehri Seyidbeyli, Ulduza Gurbanova

**Affiliations:** 1 Genetic Resources Institute, Ministry of Science and Education of the Azerbaijan Republic, Baku AZ1106, Azerbaijan; metanetbabayeva38@gmail.com (M.B.); shamsiye@bk.ru (S.M.); 2 Western Caspian University, Baku AZ1001, Azerbaijan; 3 Nakhchivan State University, Nakhchivan AZ7012, Azerbaijan; azizahuseynova@ndu.edu.az; 4 Institute of Molecular Biology, Ministry of Science and Education of the Republic of Azerbaijan, Baku AZ1073, Azerbaijan; a.pashayeva@imbb.science.az (A.P.); t.isgandarova@imbb.science.az (T.I.); s.zulfugarova@imbb.science.az (S.Z.); 5 Baku Engineering University, Khirdalan City, Hasan Aliyev str., Absheron AZ0101, Azerbaijan; 6 Azerbaijan Medical University, Baku AZ1022, Azerbaijan; mseyidbeyli@amu.edu.az

**Keywords:** synthetic wheat, drought-adapted genotypes, grain protein content, lysine, tryptophan, agro-morphological traits

## Abstract

This study was conducted to evaluate yield components, biochemical traits, and drought tolerance in 66 synthetic hexaploid wheat (SHW) genotypes and standard cultivar Karahan. The experiment was conducted over three growing seasons at the Absheron Scientific Research Base of the Institute of Genetic Resources. Genotypes were grown under irrigated and rainfed conditions using a randomised complete block design with three replications. Drought response was assessed from grain yield under irrigated and rainfed conditions using the stress tolerance index (STI), mean productivity (MP), geometric mean productivity (GMP), tolerance index (TOL), and stress susceptibility index (SSI). Genotypes combining high MP, GMP, and STI values with favourable stress-response characteristics were considered promising under water-limited conditions. Significant differences were observed among the evaluated genotypes for yield-related and biochemical traits under contrasting water regimes. Among the evaluated synthetic wheat genotypes, genotypes Sb7, Sb8, Sb9, Sb29, Sb30, andSb40 exhibited promising performance based on the combined interpretation of MP, GMP, and STI. These results suggest that these genotypes were able to maintain higher productivity under both favourable and water-limited conditions. Therefore, the combined evaluation of MP, GMP, and STI provided a more comprehensive and reliable approach for identifying promising synthetic wheat genotypes compared with the use of individual drought tolerance indices.

## 1. Introduction

Drought is among the major environmental constraints affecting agricultural productivity, food security, and water availability. Increasing drought frequency and severity have intensified agricultural challenges worldwide [1,2]. In Azerbaijan, drought and salinisation affect agricultural production, where high temperatures and insufficient rainfall reduce soil water availability and limit crop productivity [3,4].

Climate-induced reductions in soil water availability restrict water uptake and decrease cell turgor, stomatal conductance, and photosynthetic activity, ultimately affecting plant growth and grain yield. The severity of yield losses depends on drought intensity, duration, developmental stage, genotype, and environmental conditions [5,6]. Therefore, the identification of genetically diverse wheat germplasm capable of maintaining productivity under water-limited conditions is a major objective of modern breeding programmes. Consequently, the integration of morphological and biochemical traits provides valuable information for evaluating drought resilience.

Although wheat (*Triticum aestivum* L.) is a major crop for global food security, the limited diversity of the D genome in modern bread wheat restricts further improvement through conventional breeding. SHW provides an opportunity to introduce previously underutilised genetic variation from *Aegilops tauschii*, the D-genome donor of bread wheat, into breeding populations [7,8,9].

Bread wheat (*Triticum aestivum* L.; 2n = 6x = 42, AABBDD) is an allohexaploid that originated through hybridisation between a tetraploid wheat carrying the AABB genomes and *Aegilops tauschii* Coss. (2n = 2x = 14, DD), followed by chromosome doubling. SHW are artificially reconstructed through crosses between tetraploid wheat, commonly *Triticum turgidum* subsp. *durum* (Desf.) Husn. (2n = 4x = 28, AABB), and genetically diverse accessions of *Aegilops tauschii*. This approach recreates the genomic constitution of bread wheat while capturing a broader range of D-genome diversity than is generally represented in cultivated germplasm [10].

SHW and SHW-derived lines represent valuable sources of allelic variation for improving wheat adaptation, productivity, and resistance to biotic and abiotic stresses. Their primary value lies in introducing useful genetic variation into bread wheat breeding programmes rather than their direct use as commercial cultivars. Previous studies have demonstrated their potential for improving yield-related traits and tolerance when used as parental material in targeted crossing programmes [9,11]. Consequently, SHW-derived germplasm has been increasingly incorporated into breeding programmes to broaden wheat genetic diversity and improve adaptation to changing environmental conditions [12,13].

In addition to stress adaptation, SHW genotypes may exhibit valuable variation in grain-quality traits, including grain protein content and amino-acid composition. However, these characteristics vary among genotypes and require individual evaluation. Such diversity provides opportunities for simultaneous improvement of stress tolerance, productivity, and grain nutritional quality [14]. Therefore, SHW germplasm represents an important genetic resource for broadening the genetic base of bread wheat and developing productive, high-quality, and stress-adapted cultivars [12,13,15].

Physiological and biochemical traits provide valuable indicators of genotypes with improved performance under water limitation by reflecting plant water status, photosynthetic activity, and metabolic responses under water limitation [16]. Alterations in amino-acid metabolism and grain protein accumulation have been associated with drought adaptation, suggesting their potential value in evaluating stress responses [11,17,18].

Despite the recognised value of SHW germplasm, the combined agro-morphological, yield-related, biochemical, and grain-quality responses of diverse SHW genotypes under contrasting water regimes in semi-arid environments of Azerbaijan remain insufficiently characterised. Since drought responses are influenced by genotype and environmental conditions, local evaluation is essential for identifying promising genetic resources. Therefore, this study aimed to characterise SHW germplasm under irrigated and rainfed conditions in Azerbaijan by evaluating agro-morphological, biochemical, and grain-quality traits and identifying promising genetic resources for drought-adaptation breeding.

We hypothesised that SHW-derived genotypes carrying diverse *Aegilops tauschii* D-genome variation maintain higher yield stability and grain nutritional quality under drought stress compared with an adapted bread wheat cultivar.

## 2. Results

Phenotypic variability among genotypes provided an important basis for identifying promising materials for wheat improvement. Descriptive statistical parameters allowed the magnitude and distribution of variation in agronomic and biochemical traits to be assessed. Accordingly, the evaluated traits were analysed using their mean, standard deviation (SD), standard error (SE), range, and coefficient of variation (CV%). The mean values, SD, SE, and coefficient of variation (CV%) for the traits studied are presented in Table 1.

Considerable phenotypic variation was observed among the studied traits, as indicated by the range, SD, and CV%. The mean values varied from 0.696% for tryptophan content to 116.06 cm for plant height. The CV% revealed different levels of phenotypic variability among the evaluated traits. The highest relative variability was observed for unfilled grains (approximately 69.3%), followed by lysine content (approximately 22.8%) and tryptophan content (approximately 19.7%). In contrast, traits such as plant height (16.6%), peduncle length (16.6%), biomass (15.8%), thousand-kernel weight (12.4%), and grain protein content (approximately 12.0%) showed comparatively lower coefficients of variation, suggesting greater phenotypic stability across the evaluated genotypes.

Among agronomic traits, plant height showed a mean value of 116.06 cm with a SD of 19.30 cm and a CV of 16.63%. Thousand-kernel weight averaged 45.12 g, with a SD of 5.60 g and a CV of 12.42%, indicating relatively low variability for this important yield component. Biomass showed a mean value of 4.56 with a SD of approximately 0.72 and a CV of 15.8%. Spike-related traits also demonstrated variation among genotypes, reflecting differences in morphological characteristics.

Grain-quality traits exhibited variable levels of phenotypic diversity. Grain protein content showed a mean value of approximately 14.42% with a CV of about 12.0%. Lysine content had a mean value of approximately 1.81% and a CV of about 22.8%. Tryptophan content showed a mean value of approximately 0.70% and exhibited moderate variability among the evaluated genotypes. Overall, the observed variation in agronomic and grain-quality traits indicates substantial genetic diversity within the synthetic wheat genotypes and provides useful variation for selection and breeding programs.

Considerable phenotypic variability was observed among the evaluated SHW genotypes. Plant height varied from 67.0 to 170.0 cm, while thousand-kernel weight ranged from 18.0 to 61.38 g. Grain biochemical traits, including grain protein content, lysine, and tryptophan contents, exhibited moderate variation among genotypes. The observed variability indicated that the evaluated germplasm contained valuable diversity for agronomic performance and grain-quality traits. However, confirmation of significant genotypic differences required variance analysis based on plot-level observations.

Since the analysis of variance revealed significant genotype effects for several agronomic and grain-quality traits, Tukey’s honestly significant difference (HSD) test was performed to compare mean differences among synthetic wheat genotypes. Significant variation among genotypes was observed for plant height, peduncle length, flag leaf length, biomass, spike characteristics, grain yield, and grain-quality traits. Genotypes belonging to different statistical groups demonstrated distinct phenotypic performance, indicating substantial genetic diversity within the evaluated synthetic wheat collection. Several genotypes, including Sb7, Sb8, Sb9, Sb30, and Sb40 showed favourable mean performance across yield-related traits and drought tolerance indices, although statistical grouping varied depending on the evaluated trait (Table S1).

Combined mixed-model analysis of variance revealed significant effects of genotype (G), water regime/environment (W), growing season (Y), and their interactions on several agronomic and grain-quality traits (Table 2). The magnitude and significance of these effects varied among traits, indicating differential genetic control and environmental responsiveness of the evaluated characteristics.

The genotype effect was highly significant (*p* ≤ 0.001) for plant height, peduncle length, flag-leaf length, biomass, unfilled grains, thousand-kernel weight, spikelets per spike, grain protein content, and lysine content, demonstrating substantial genetic variation among the evaluated synthetic wheat genotypes. In contrast, genotype effects were not significant for spike length, spike weight, and tryptophan content, suggesting relatively lower genetic differentiation for these traits under the tested conditions.

Growing season significantly affected most agronomic traits, including plant height, peduncle length, flag-leaf length, biomass, unfilled grains, thousand-kernel weight, and spikelets per spike. The strongest seasonal effects were observed for unfilled grains (F = 1549.46 ***), plant height (F = 589.80 ***), biomass (F = 317.23 ***), and flag-leaf length (F = 161.81 ***), indicating considerable interannual environmental variation. In contrast, growing season effects were not significant for grain protein content, lysine content, spike length, spike weight, and tryptophan content.

Significant G × W interactions were detected for plant height, peduncle length, flag-leaf length, biomass, unfilled grains, thousand-kernel weight, spikelets per spike, grain protein content, and lysine content. These interactions indicate that genotypic responses differed between water regime, suggesting the presence of suggesting differential responses among genotypes. However, G × W interactions were not significant for spike length, spike weight, and tryptophan content.

G × Y interactions were significant for plant height, peduncle length, flag-leaf length, biomass, unfilled grains, and spikelets per spike. Additionally, lysine content showed a significant G × Y interaction (F = 1.63 ***). In contrast, G × Y effects were not significant for thousand-kernel weight, spike length, spike weight, grain protein content, and tryptophan content.

W × Y interactions were significant for plant height, peduncle length, flag-leaf length, biomass, unfilled grains, thousand-kernel weight, spikelets per spike, and lysine content. The strongest W × Y effects were observed for plant height (F = 666.56 ***), biomass (F = 463.22 ***), unfilled grains (F = 59.26 ***), and spikelets per spike (F = 39.07 ***), indicating that environmental responses varied between growing seasons.

The three-way interaction (G × W × Y) was significant for plant height, peduncle length, flag-leaf length, biomass, unfilled grains, spikelets per spike, and lysine content. These results demonstrate that genotype performance under contrasting water regimes was not completely consistent across growing seasons, highlighting the importance of evaluating drought responses across multiple environments and years. Conversely, G × W × Y interactions were not significant for thousand-kernel weight, spike length, spike weight, grain protein content, and tryptophan content.

Overall, the mixed-model analysis confirmed significant genetic and environmental contributions to variation in most agronomic traits and selected grain-quality characteristics. The presence of significant genotype × environment interactions indicates that genotype selection should consider both productivity potential and environmental adaptability, particularly under contrasting water availability conditions.

Significant G × W interactions were observed for plant height, biomass, unfilled grains, thousand-kernel weight, spikelets per spike, grain protein content, lysine content, and grain yield, indicating differential genotype responses across irrigated and rainfed conditions. Significant G × Y and W × Y interactions for several agronomic traits demonstrated that genotype performance varied among growing seasons and that environmental effects differed across years. Moreover, significant G × W × Y interactions for plant height, peduncle length, flag-leaf length, biomass, unfilled grains, spikelets per spike, lysine content, and grain yield indicated that genotype responses were influenced by the combined effects of water regime and growing season.

Grain yield performance under irrigated and rainfed conditions was used to calculate drought tolerance indices, including MP, GMP, TOL, SSI, and STI (Table 3 and Table S2). Significant variation was observed among synthetic wheat genotypes for yield performance and drought response parameters. Genotypes Sb7, Sb8, Sb9, Sb29, Sb30 and Sb40 showed comparatively favourable combinations of MP, GMP, and STI values, indicating stable yield performance under contrasting water regimes.

Among yield-related traits, grain yield exhibited significant effects of genotype, water regime, growing season, and their interaction components, highlighting the contribution of genetic and environmental factors to yield variation. In contrast, spike length, spike weight, and tryptophan content showed predominantly non-significant responses, suggesting relatively lower variation among tested environments and genotypes for these traits.

The mixed-model analysis of variance was conducted to partition the sources of variation associated with G, W, Y and their interactions for grain yield and related traits. Although analysis of variance (ANOVA) identifies the significance of genotype and environmental effects, it does not provide direct information on genotype performance patterns and stability across multiple environments. Therefore, GGE biplot analysis was performed to evaluate genotype performance and stability across six environments defined by the combination of three growing seasons (2022–2023, 2023–2024, and 2024–2025) and two water regimes (irrigated and rainfed conditions). The GGE approach integrates genotype main effects and genotype × environment interaction effects, enabling the identification of genotypes with superior mean performance and stable yield expression across contrasting environments.

**Figure 1:**
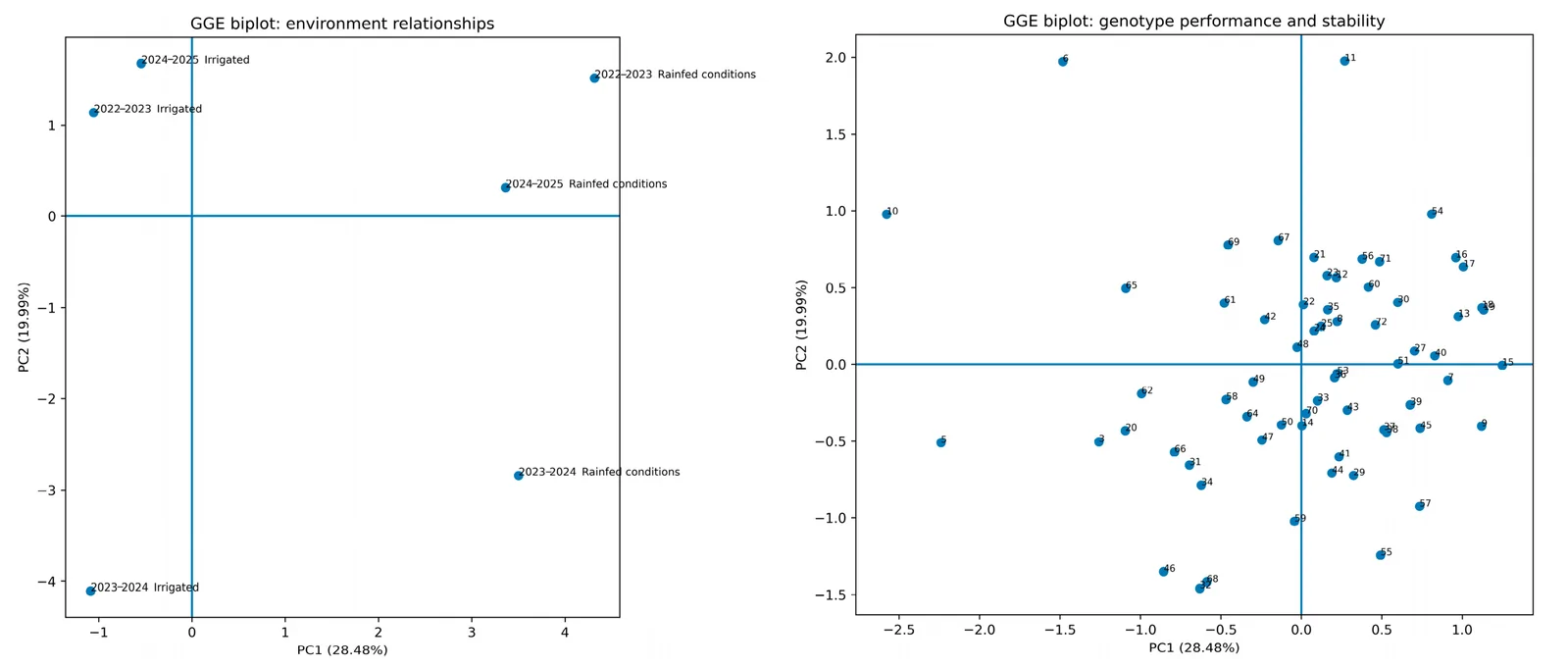
Multi-year GGE biplot analysis of genotype × environment interaction across year × water regime combinations for grain yield in SHW genotypes. Dots represent genotype or environment scores, and numbers in the genotype performance and stability biplot indicate genotype identifiers.

In the present study, GGE biplot analysis based on grain yield revealed the contribution of genotype and genotype × environment effects to performance differentiation among synthetic wheat genotypes. The first two principal components explained a substantial proportion of the total variation, providing a graphical representation of genotype stability and environmental relationships (Figure 1).

Correlation analysis revealed a weak negative relationship between grain yield under irrigated (Yp) and rainfed conditions (Ys) (r = −0.137, *p* > 0.05), indicating that yield performance under irrigated conditions was not strongly associated with yield performance under rainfed conditions (Table 4).

Pearson correlation analysis revealed significant relationships among yield performance under contrasting water regimes and drought tolerance indices (Table 4). Grain yield under irrigated conditions (Yp) showed significant positive correlations with TOL (r = 0.679, *p* < 0.01), MP (r = 0.544, *p* < 0.01), GMP (r = 0.524, *p* < 0.01), and STI (r = 0.531, *p* < 0.01). These associations indicate that genotypes exhibiting higher yield potential under favourable conditions generally showed higher values for productivity-related indices. Yield under rainfed conditions (Ys) showed strong positive correlations with MP (r = 0.757, *p* < 0.01), GMP (r = 0.768, *p* < 0.01), SSI (r = 0.823, *p* < 0.01), and STI (r = 0.759, *p* < 0.01). These indices reflected different aspects of genotype performance and stress response under water-limited conditions. The strongest associations were observed between MP and GMP (r = 0.997, *p* < 0.01), and between GMP and STI (r = 0.997, *p* < 0.01), indicating a high degree of consistency among these indices for evaluating drought response and yield stability.

In contrast, the strong positive relationships of MP, GMP, and STI with Ys indicate that these indices may be more effective for identifying genotypes combining productivity and drought tolerance.

Although MP and GMP showed a very strong correlation, their similar relationships with Yp and Ys suggest that both indices provide comparable information regarding genotype ranking across contrasting environments. Therefore, either MP or GMP, together with STI, may serve as useful selection criteria for identifying synthetic wheat genotypes with stable yield performance under drought stress. Principal component analysis based on twelve agronomic and grain-quality traits revealed the contribution of individual traits to genotype differentiation (Table 5).

**Figure 2:**
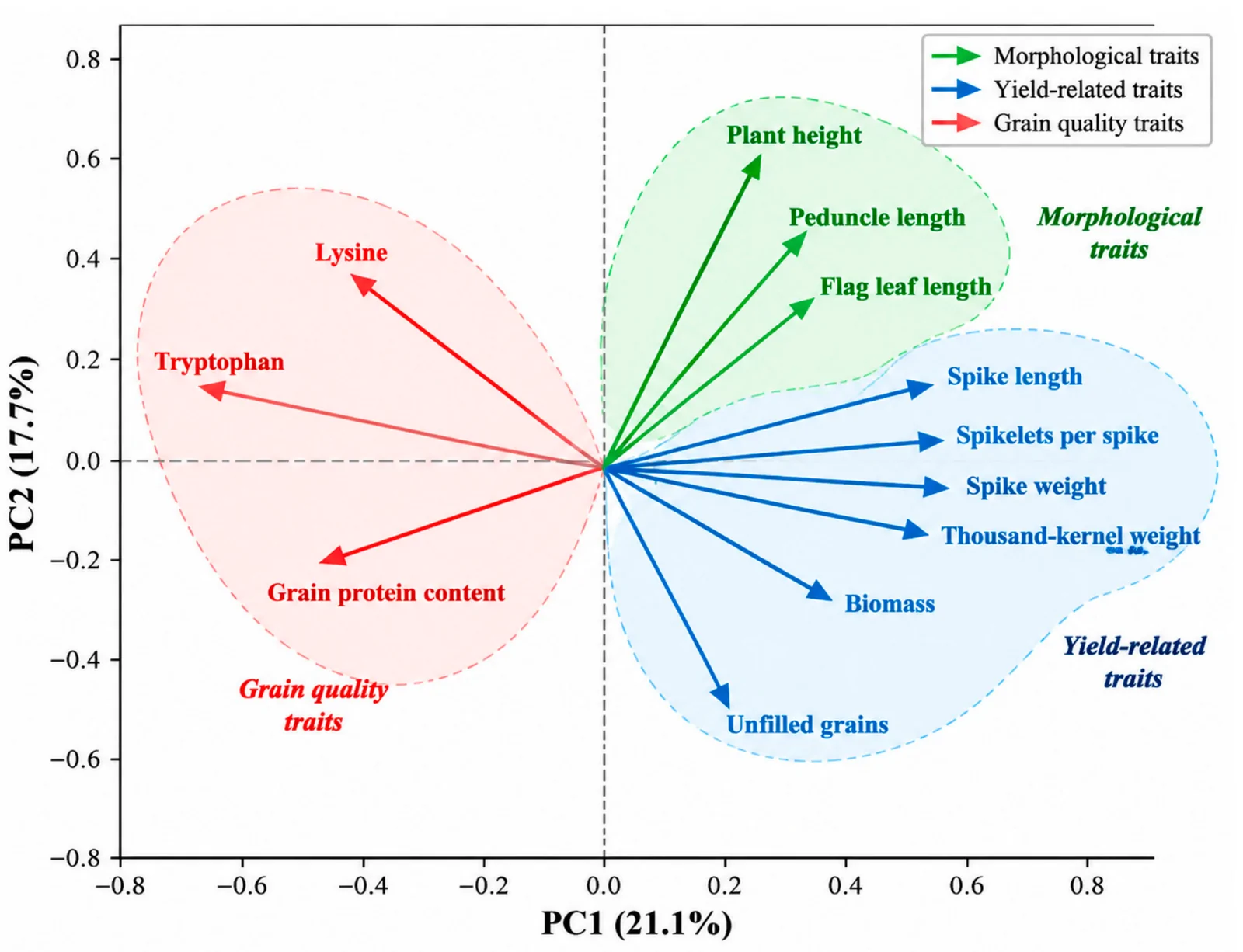
Principal component analysis (PCA) loading plot showing the relationships among agronomic and grain-quality traits in synthetic wheat genotypes.

The contrasting loadings of protein, lysine, and tryptophan on PC1 suggested the presence of potential different contribution patterns among the evaluated grain-quality characteristics. Meanwhile, the simultaneous contribution of plant height and peduncle length to PC2 indicated that plant architectural traits contributed to genotype differentiation. Associations among traits assessed through principal component analysis may reflect shared genetic and physiological influences among evaluated traits; however, the underlying mechanisms require further investigation (Figure 2). The PCA was performed using standardised quantitative traits after z-score transformation. The arrows represent the direction and magnitude of trait contributions to the first two principal components. The angle between vectors indicates the relationship among traits, with smaller angles representing positive associations and opposite directions indicating negative relationships. The first two principal components (PC1 and PC2) explain the major proportion of total phenotypic variation, with the explained variance (%) indicated in parentheses. Trait groups are visually categorised as morphological, yield-related, and grain-quality characteristics.

The first principal component (PC1) explained 21.1% of the total variation and mainly separated genotypes based on the contrast between yield-related/morphological performance and grain-quality characteristics. Positive contributions along PC1 were mainly associated with plant height, biomass, spikelets per spike, spike length, spike weight, and thousand-kernel weight, indicating that these traits contributed strongly to plant productivity and yield formation. In contrast, grain-quality traits, particularly lysine, tryptophan, and grain protein content, showed negative associations with PC1, suggesting differentiation between productivity-related traits and grain nutritional attributes.

The second principal component (PC2) accounted for 17.7% of the total variation and was mainly associated with morphological characteristics and grain-quality variation. Plant height, peduncle length, and flag leaf length showed strong positive contributions to PC2, indicating that plant architecture was an important source of phenotypic differentiation. Meanwhile, lysine and tryptophan contents contributed negatively/positively along this axis, reflecting variation in grain nutritional composition.

Yield-related traits showed strong relationships within the PCA space. Spike length, spikelets per spike, spike weight, and thousand-kernel weight were positioned in a similar direction, indicating positive associations among these yield components. Biomass also showed a positive relationship with yield-related traits, suggesting that greater vegetative growth was associated with improved productivity characteristics.

Grain-quality traits formed a separate group in the PCA biplot. Protein content, lysine, and tryptophan showed distinct contributions compared with morphological and yield-related traits, indicating that nutritional quality represented an independent source of variation among the evaluated genotypes.

The PCA results confirm that synthetic wheat genotypes possess considerable diversity for both agronomic performance and grain nutritional characteristics. The separation of morphological, yield-related, and grain-quality traits indicates that selection strategies should consider multiple trait combinations rather than individual characteristics alone.

**Figure 3:**
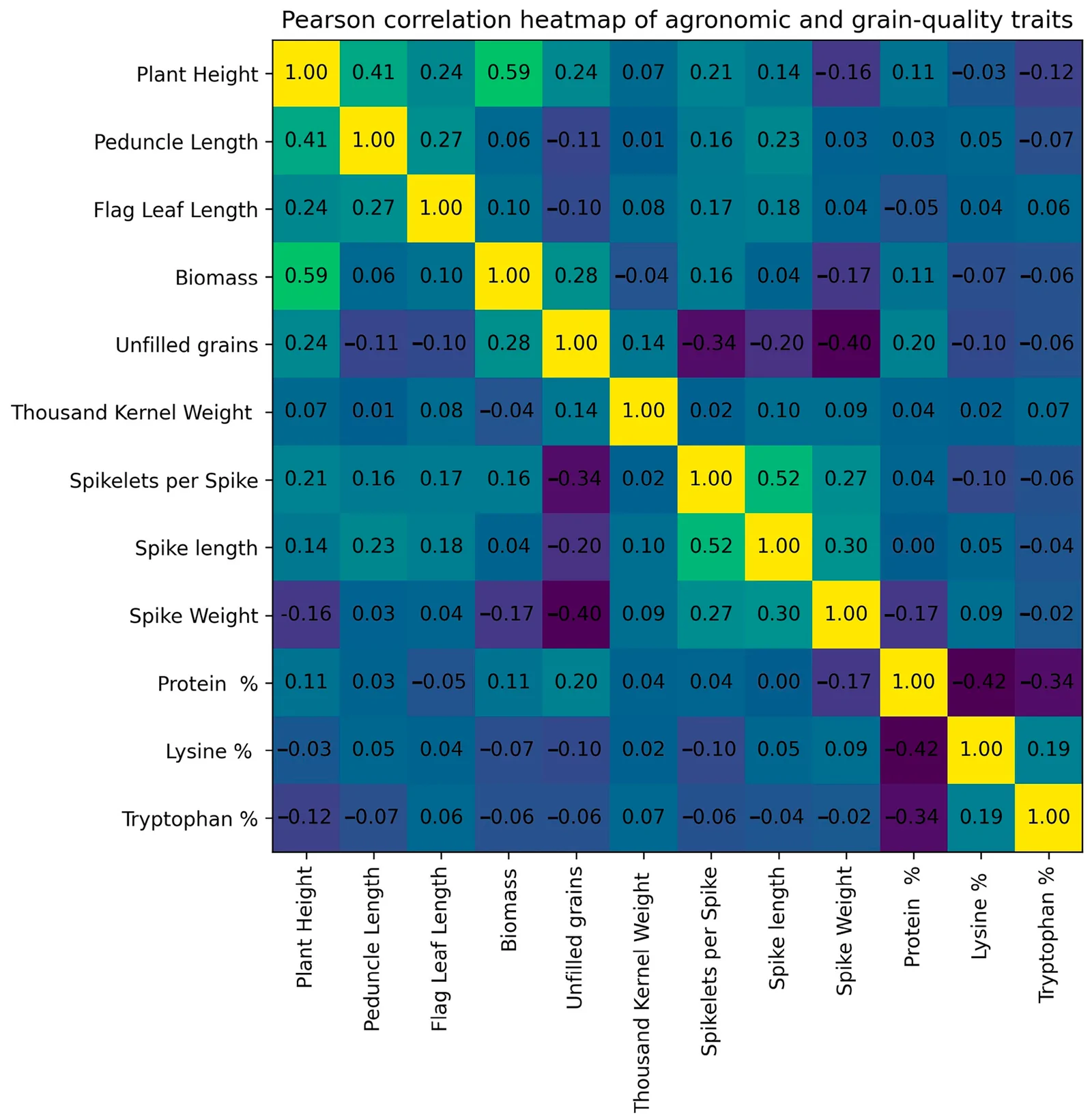
Pearson correlation heatmap showing relationships among agronomic, yield-related, and grain-quality traits in synthetic wheat genotypes. Yellow shades indicate positive correlations, with increasing intensity reflecting stronger associations, whereas dark blue to purple shades represent negative correlations. Intermediate colors (green to light blue) correspond to weak to moderate correlations.

By identifying traits that were significantly correlated, it was possible to identify significant relationships among traits, which may facilitate the selection of associated characteristics. Yu et al. (2020) [18]. reported a positive and significant correlation between plant height and spike length. Their study suggested that taller genotypes tended to rely more on the supply of photosynthetic materials [19]. Pearson correlation analysis revealed significant associations among morphological traits, drought tolerance indices, and grain-quality characteristics (Figure 3).

Plant height was positively correlated with biomass (r = 0.591), indicating that taller plants tended to exhibit greater biomass accumulation. However, biomass showed weak correlations with yield-related traits, including spike weight (r = −0.164) and thousand-kernel weight (r = 0.073), suggesting that variation in vegetative growth was not consistently associated with changes in these yield components.

Grain protein content exhibited significant negative correlations with lysine (r = −0.418, *p* < 0.01) and tryptophan (r = −0.343, *p* < 0.01), indicating an inverse relationship among these grain nutritional components. These associations suggest that variation in protein accumulation may influence the relative distribution of amino acid components; however, the observed relationships should be interpreted as trait associations rather than direct causal effects.

Overall, the observed correlation pattern among yield-related traits and drought tolerance indices supports the use of multiple selection criteria for identifying synthetic wheat genotypes with favourable yield performance under contrasting water regimes.

## 3. Discussion

Drought stress represents one of the most important abiotic constraints limiting wheat production, particularly under rainfed conditions where insufficient and irregular precipitation restricts plant growth, reproductive development, and grain formation. Given the importance of wheat (*Triticum aestivum* L.) for global food security, identifying genotypes with stable performance under contrasting water regimes is essential for breeding programmes aimed at developing climate-resilient cultivars. Identifying genotypes with favourable performance under water-limited conditions requires integrating yield-based indicators with agronomic traits that reflect genotype responses under contrasting environments. In addition to yield-related characteristics, drought stress induces complex physiological and biochemical changes in wheat, including alterations in antioxidant activity, protein metabolism, and cellular stability, which contribute to variation in stress responses among genotypes [20]. Previous studies have shown that morphological stress symptoms, such as leaf tip drying and leaf rolling, may be associated with reduced performance and increased sensitivity to water limitation [21].

SHW germplasm developed through CIMMYT and ICARDA breeding programmes represents an important source of phenotypic diversity for improving wheat performance under drought-prone environments. Previous studies conducted under semi-arid and water-limited environments have demonstrated that SHW-derived materials can contribute favourable alleles associated with yield stability, stress tolerance, and improved agronomic performance. Consistent with these reports, the present study revealed considerable variation among SHW genotypes in productivity-related drought tolerance indices and grain-related traits under contrasting water regimes. Similar approaches combining multi-trait evaluation under water-limited environments have recently been applied to identify promising wheat genotypes with improved drought tolerance and yield stability [22].

Genotypes Sb7, Sb8, Sb9, Sb29, Sb30, and Sb40 showed relatively favourable values of MP, GMP, and STI, indicating their ability to maintain high grain yield performance across contrasting water regimes. (Table 3).

The identification of promising genotypes was based on the combined evaluation of multiple drought tolerance indices rather than a single parameter. Although indices such as TOL and SSI provide information regarding yield reduction and stress sensitivity, they may not fully reflect overall genotype performance because they can favour genotypes with lower yield potential.

The significant effects of growing season and genotype × environment interactions demonstrated that environmental variation influenced genotype performance across years. Consequently, genotype evaluation based on multi-year and multi-environment performance provides a more reliable assessment of drought tolerance and yield stability than single-season evaluation. The consistent performance of high-ranking genotypes across contrasting environments highlights their potential value for further breeding investigations. Therefore, simultaneous consideration of yield under stress conditions, MP, GMP, STI, and other productivity-related parameters provides a more balanced strategy for identifying genotypes combining drought tolerance index performance with high productivity [23].

Correlation analysis supported the importance of productivity-related drought tolerance indices for evaluating synthetic wheat genotypes (Table 4). Yield under irrigated conditions (Yp) showed positive correlations with MP (r = 0.544, *p* < 0.01), GMP (r = 0.524, *p* < 0.01), and STI (r = 0.531, *p* < 0.01), whereas yield under rainfed conditions (Ys) exhibited stronger positive correlations with MP (r = 0.757, *p* < 0.01), GMP (r = 0.768, *p* < 0.01), and STI (r = 0.759, *p* < 0.01). These results indicate that MP, GMP, and STI were strongly associated with yield performance under contrasting water regimes and may serve as useful selection criteria for identifying synthetic wheat genotypes with favourable drought tolerance index profiles.

The strongest correlation was observed between MP and GMP (r = 0.997, *p* < 0.01), followed by the relationship between GMP and STI (r = 0.997, *p* < 0.01), demonstrating high consistency among these indices for genotype ranking. Similar relationships among productivity-based drought indices have been reported in wheat. Rezaei (2026) [24] reported significant associations between yield performance and drought tolerance indices in soft wheat genotypes, while Bhandari et al. (2024) [25] demonstrated strong relationships among MP, GMP, and STI, supporting the usefulness of these indices for identifying genotypes with stable yield performance under water-limited conditions [26].

In contrast, TOL showed a positive relationship with Yp (r = 0.679, *p* < 0.01) but a strong negative association with Ys (r = −0.820, *p* < 0.01) and SSI (r = −0.995, *p* < 0.01). These results suggest that TOL mainly reflects yield differences between contrasting environments and should therefore not be used independently for genotype selection. The combined application of multiple yield-based indices provides a more comprehensive approach for identifying genotypes with favourable drought tolerance index profiles and balanced productivity under stress conditions. Beyond yield-based indicators, grain-quality traits contributed to genotype differentiation under contrasting environments. Protein, lysine, and tryptophan contents showed variation among synthetic wheat genotypes, indicating diversity in grain nutritional characteristics. However, the physiological and biochemical mechanisms responsible for changes in grain-quality traits under drought conditions were not directly evaluated in this study. Future investigations incorporating nitrogen metabolism, carbon partitioning, and physiological stress responses may provide further insights into the regulation of grain quality under water limitation. Morphological and yield-related traits, particularly spike characteristics, also contributed to genotype differentiation. Maintenance of reproductive traits under water-limited conditions is important because grain formation is highly sensitive to drought stress. The integration of spike-related traits with productivity-based drought indices and grain-quality characteristics may therefore provide a more effective strategy for identifying synthetic wheat genotypes with improved agronomic performance under variable environments [27].

Synthetic wheat materials represent valuable genetic resources for improving drought resilience and yield stability in wheat breeding programs. Their broad genetic background provides opportunities for introducing and recombining favourable alleles from wild relatives and diverse germplasm sources [9,28]. This expanded genetic diversity may facilitate the development of genotypes combining high productivity, drought tolerance and improved grain nutritional quality under future climate scenarios.

## 4. Materials and Methods

### 4.1. Plant Materials

A total of 66 SHW genotypes and standard cultivar Karahan were used in this study. These materials were developed by incorporating diverse *Aegilops tauschii* D-genome variation into cultivated wheat backgrounds and represent a broad genetic resource for improving adaptation-related traits in bread wheat.

The evaluated SHW lines originated from different pedigree backgrounds, including combinations involving *Aegilops tauschii* accessions and elite durum wheat-derived parents. Detailed pedigree information, available donor accessions, and genetic background of the evaluated materials are provided in Table 6.

The synthetic wheat collection was selected because of its potential to provide novel allelic variation associated with adaptation to abiotic stresses, yield stability, and grain quality improvement. The diversity within these materials enables comparative assessment of the contribution of *Aegilops tauschii*-derived variation to wheat performance under contrasting environmental conditions.

In addition to the SHW-derived lines, the modern bread wheat cultivar Karahan was included as a reference check genotype. Karahan was selected because it represents an adapted bread wheat background and has been used as a comparison genotype in wheat improvement studies. Its inclusion allowed evaluation of whether synthetic-derived materials carrying diverse *Aegilops tauschii*-derived D-genome variation could provide advantages over conventional bread wheat germplasm.

### 4.2. Experimental Design and Field Conditions

The field experiments were conducted during the 2022–2025 growing seasons under two contrasting water regimes: irrigated and rainfed conditions. *The two water environments were established as separate but adjacent field experiments at the same experimental station. During each growing season, each water environment was arranged independently as a randomised complete block design (RCBD) with three replications.* A total of 66 synthetic wheat genotypes and standard cultivar Karahan were evaluated. Within each block, the genotypes were randomly assigned to individual plots to minimise the effects of spatial heterogeneity across the experimental field. *Each genotype was represented once per block, resulting in three independent replicate plots per genotype within each water environment. Individual plots were considered the experimental units for all statistical analyses.* Each plot consisted of six rows, 5 m in length, with 20 cm spacing between rows, corresponding to a total plot area of 6 m^2^. To minimise potential border effects, the outer rows were excluded from sampling, and all agronomic measurements were performed using plants from the central rows.

The study was conducted at the Absheron Scientific Research Base Experimental Station of the Institute of Genetic Resources. In the study area, the average temperature for January during 2022–2025 was 3.8 °C, and for July, it was 22.6 °C. Annual precipitation in Absheron ranged between 180 and 260 mm, and most rainfall occurred during the colder periods. Humidity levels did not change throughout the year. No significant deviations from the long-term climate indicators appeared during the study years. Climatic data, including maximum, minimum, and mean temperatures as well as total precipitation for the 2022–2025 growing seasons, are provided in Table S3.

The irrigated treatment received five supplemental irrigations during the growing season. Irrigation events were applied at critical phenological stages of winter wheat, including tillering, stem elongation, flag leaf emergence, flowering, and grain filling, to maintain adequate soil moisture and prevent water limitation. The irrigated treatment received five supplemental irrigations during the growing season. Details of irrigation timing, crop growth stages, and applied water amounts are provided in Table S4.

### 4.3. Soil Characteristics

The soil at the experimental site belongs to the light chestnut (calcareous) soil type. It is slightly alkaline, with medium humus content, low available phosphorus, and normal potassium levels. In the arable layer, pH was 8.6, humus content ranged between 1.25 and 2.17%, and 1 kg of soil contained 42.7 mg of available nitrogen, 23.4 mg of phosphorus, and 170.5 mg of K_2_O. Before sowing, 55 kg ha^−1^ of phosphorus (P) and 40 kg ha^−1^ of potassium (K) fertilizers were applied as active ingredients, while 70 kg ha^−1^ of nitrogen (N) fertilizer was applied as top dressing in spring.

Soil water content was monitored at 0–30, 30–60, and 60–90 cm depths throughout the season. Field capacity (FC) was determined for each horizon, and volumetric water content (θ) was measured at three time points to capture progressive soil drying (Supplementary Table S4). Border rows were excluded from sampling to minimise edge effects and improve the accuracy of field measurements.

### 4.4. Measurement of Agronomic Traits

Agronomic and yield-related traits were evaluated at physiological maturity for each genotype under both irrigated and rainfed conditions. Ten representative plants were randomly selected from the central rows of each replicate plot, excluding border plants, and used for individual plant measurements. Measurements obtained from individual plants within each plot were averaged to generate a single plot-level value for statistical analyses.

Plant height (cm) was measured from the soil surface to the tip of the main spike, excluding awns, at physiological maturity. Peduncle length (cm) was determined as the length of the uppermost internode between the flag leaf node and the base of the spike. Flag leaf length (cm) was measured from the ligule to the tip of the flag leaf at the flowering stage.

Biomass was determined by harvesting above-ground plant material from each plot, followed by oven drying until constant weight was achieved. The dry biomass was recorded as the total above-ground biomass per plot.

Yield-related traits were measured using representative spikes collected from each plot. Spike length (cm) was measured from the base of the spike to the tip, excluding awns. Spikelets per spike were determined by manually counting the number of spikelets on each sampled spike. Spike weight (g) was recorded by weighing individual spikes after harvest.

Unfilled grains were determined after threshing sampled spikes by separating undeveloped grains from fully developed grains and recording the number of unfilled grains per spike. Thousand-kernel weight (TKW, g) was measured by randomly selecting and weighing 1000 fully developed kernels using a precision balance. Grain yield (t ha^−1^) was determined by harvesting the central area of each plot, adjusting grain moisture to the standard level, and converting the harvested grain weight to yield per hectare.

### 4.5. Determination of Biochemical Parameters

#### 4.5.1. Determination of Total Grain Protein Content

Total protein content in synthetic wheat grains was determined using the Kjeldahl method. 100 mg of finely ground wheat flour was transferred to a 100 mL Kjeldahl digestion flask, followed by the addition of 7 mL of concentrated sulfuric acid (H_2_SO_4_) (Honeywell Research Chemicals, Germany). A selenium–copper mixture was used as the digestion catalyst.

The samples were digested in a fume hood until a clear solution was obtained. After cooling, the inner walls of the flask were rinsed with distilled water, and the samples were heated for an additional 10 min to ensure complete digestion. Subsequently, the digested samples were subjected to distillation using a Kjeldahl apparatus.

For ammonia collection, 25 mL of 0.02 N H_2_SO_4_ (Honeywell Research Chemicals, Germany) and 1–2 drops of indicator were added to each 200 mL receiving flask. The digestion flasks were connected to the Kjeldahl apparatus, and the digested samples were alkalised with a 40% alkaline solution. Distillation was continued for 20–30 min. The solution collected in the receiving flask was then back-titrated with 0.02 N NaOH (Sigma-Aldrich, St. Louis, MO, USA) until the endpoint, indicated by a distinct colour change, was reached.

The total protein content was calculated according to the following equation:

$$\mathrm{Protein}\mathrm{content}\left(\%\right)=\frac{\left(25-V\right)\times 0.28\times 100}{m}\times \frac{100}{89}\times 6.25$$

where *V* is the volume of 0.02 N NaOH (Sigma-Aldrich, St. Louis, MO, USA) used for back-titration (mL); 25 is the initial volume of 0.02 N H_2_SO_4_ (Honeywell Research Chemicals, Germany) added to the receiving flask (mL); 0.28 is the nitrogen-equivalent coefficient for 0.02 N H_2_SO_4_ (Honeywell Research Chemicals, Germany); m is the mass of the analysed sample; 100/89 is the dry-matter correction factor; and 6.25 is the nitrogen-to-protein conversion factor [29].

#### 4.5.2. Determination of Lysine Content

Lysine content in synthetic wheat grains was determined using a ninhydrin-based colorimetric method. Briefly, 30 mg of finely ground wheat flour was weighed into a 100 mL flask in duplicate. Subsequently, 1 mL of 2% Na_2_CO_3_ (Sigma-Aldrich, St. Louis, MO, USA) solution and 250 mg of finely powdered glass were added. The mixture was thoroughly homogenised using a glass rod and incubated in a water bath at 80 °C for 10 min.

After incubation, the samples were removed from the water bath, and 2 mL of ninhydrin reagent (Sigma-Aldrich, St. Louis, MO, USA) was added. For calibration, 0.5 mL of each standard solution was mixed with 0.5 mL of 4% Na_2_CO_3_ (Sigma-Aldrich, St. Louis, MO, USA) solution and 2 mL of ninhydrin reagent. Both the samples and standards were subsequently incubated in a water bath for 30 min.

Following incubation, the mixtures were cooled to room temperature, supplemented with 5 mL of 95% ethanol (Sigma-Aldrich, St. Louis, MO, USA), and centrifuged for 5 min. The absorbance of the resulting clear supernatant was measured using a photoelectric colorimeter equipped with a green filter. Lysine content was calculated using the calibration curve generated from the standard solutions.

The lysine content was calculated according to the following equation:

$$X=\frac{0.15\;\times A\;\mathrm{mean}}{A\mathrm{standard}}\times \frac{1000}{30\;\times 11}$$

where *X* represents the lysine content, *A* mean is the mean absorbance of the sample, and *A* standard is the absorbance of the standard solution closest to that of the analysed sample [30].

#### 4.5.3. Determination of Tryptophan Content

Tryptophan content in synthetic wheat grains was determined according to the colorimetric method of Ermakov and Yarosh (1969), with minor modifications. Briefly, 200 mg of finely ground wheat flour was transferred into a 100 mL volumetric flask, and 2 mL of distilled water was added. Subsequently, 1 mL of a 4% gelatin solution prepared in 25% KOH (Sigma-Aldrich, St. Louis, MO, USA) was added. The flasks were tightly sealed with rubber stoppers, and the samples were incubated at 40 °C for 18–20 h [31].

Following incubation, the samples were cooled to room temperature. Subsequently, 0.5 mL of p-dimethylaminobenzaldehyde reagent prepared in 10% HCl (Sigma-Aldrich, St. Louis, MO, USA), 0.5 mL of 1% sodium nitrate (NaNO_3_) solution HCl (Sigma-Aldrich, St. Louis, MO, USA), and 28 mL of concentrated HCl were sequentially added to each flask. The reaction mixtures were thoroughly mixed, resealed, and incubated in a thermostatically controlled chamber at 25 °C for 90 min.

After colour development, the volume of each sample was adjusted to 100 mL with distilled water. The resulting solutions were filtered, and their absorbance was measured at 590 nm against a reagent blank using a spectrophotometer (Amersham Biosciences, UK). Tryptophan concentration was determined using an external calibration curve prepared from analytical-grade L-tryptophan standards (Merck KGaA, Darmstadt, Germany) subjected to the same colour-development procedure.

Tryptophan content was calculated according to the following equation:

$$\mathrm{Tryptophan}\;\mathrm{content}\;(\mathrm{mg}\;100g^{-1})=\frac{C\;\times V\times \mathrm{DF}\times 100}{m}$$

where *C* is the tryptophan concentration obtained from the calibration curve (mg mL^−1^), *V* is the final volume of the sample solution (mL), DF is the dilution factor, and m is the mass of the analysed grain sample (g). The results are expressed as milligrams of tryptophan per 100 g of grain dry matter.

### 4.6. Genotypes with Improved Performance Under Drought Tolerance Indices

Grain yield data obtained under irrigated and rainfed conditions were used to calculate genotypes with improved performance under drought tolerance indices and to evaluate the productivity, stability, and stress response of the synthetic wheat genotypes. The corresponding mean yields of all evaluated genotypes under irrigated and rainfed conditions are denoted by Yp and Ys, respectively.

The following genotypes with improved performance under drought tolerance indices were calculated:

MP:

$$\mathrm{MP}=\frac{\mathrm{Yp}+\mathrm{Ys}}{2}$$

TOL:

TOL = Yp − Ys

GMP:

$$\mathrm{GMP}=\sqrt{\mathrm{Yp}\times \mathrm{Ys}}$$

STI:

$$\mathrm{STI}=\frac{Y_{p}\times Y_{s}}{\bar{Y_{p}^{2}}}$$

SSI:

$$\mathrm{SSI}=\frac{1-\left(Y_{s}/Y_{p}\right)}{\mathrm{SI}}$$

where Yp represents the grain yield of a genotype under irrigated conditions and Ys represents its grain yield under rainfed conditions. Lower TOL values indicate a smaller reduction in grain yield between non-stress and rainfed conditions.

Genotypes exhibiting high MP, GMP, and STI values together with low DTI and SSI values were considered to possess promising yield stability and genotypes with improved performance under water limitation. These indices have been widely used to identify high-yielding and drought-tolerant wheat genotypes under contrasting water regimes [25,32,33].

### 4.7. Statistical Analysis

The field experiments were arranged as randomised complete block designs with three replications within each water environment and growing season. Individual field plots constituted the experimental units. Measurements obtained from individual plants within the same plot were considered subsamples and were averaged to obtain a single plot-level value for each trait. For biochemical analyses, grain collected from plants within each replicate plot was pooled to obtain one composite biological sample per experimental unit. Repeated analytical measurements of the same composite sample were treated as technical replicates and averaged prior to statistical analysis. For traits evaluated across the three growing seasons and two water environments, plot-level observations were subjected to a combined mixed-model analysis of variance. The model included G, W, growing season (Y), and their interactions (G × W, G × Y, W × Y, and G × W × Y) as fixed effects. Block (replication) nested within each year × water-regime combination was included as a random effect to account for variation among blocks within experimental environments.

The statistical model was:

Trait = μ + G + W + Y + G × W + G × Y + W × Y + G × W × Y + Block(Y × W) + ε

where μ represents the overall mean and ε represents residual plot-level variation. For the tests of W, Y, and W × Y, variation among blocks nested within year × water regime was used as the corresponding error stratum, whereas genotype and genotype-associated interaction terms were tested against plot-level residual variation.

Pearson correlation analysis was performed using genotype mean values calculated across years and water regimes to avoid pseudoreplication. Each genotype was considered as an independent observation.

Model assumptions were evaluated through residual analysis for normality and homogeneity of variance. Significant differences among treatment means were separated using HSD test at *p* < 0.05.

To assess genotype performance and stability across contrasting environments, GGE biplot analysis was performed using grain yield data obtained from all genotype × environment combinations. Each combination of growing season and water regime was considered as a separate environment, resulting in six environments representing three growing seasons (2022–2023, 2023–2024, and 2024–2025) under irrigated and rainfed conditions. The GGE model was applied to partition the observed variation into genotype effects and genotype × environment interaction effects while excluding the environmental main effect. The first two principal components (PC1 and PC2) were used to construct the GGE biplot and visualise genotype performance, environmental relationships, and genotype stability across environments. The average environment coordination (AEC) approach was used to assess mean performance and stability of genotypes, whereas the “which-won-where” view was used to identify genotype responses across different environments.

## 5. Conclusions

The identification of promising synthetic wheat genotypes based on the combined evaluation of drought tolerance indices provides valuable genetic resources for developing future wheat cultivars with improved drought tolerance and stable productivity under changing climatic conditions.

The evaluated SHW genotypes exhibited considerable phenotypic variation in agronomic performance and drought response under contrasting water regimes. Among the evaluated drought tolerance indices, MP, GMP, and STI were the most informative parameters for identifying genotypes combining high yield potential with favourable performance under irrigated and rainfed conditions. In particular, genotypes Sb7, Sb8, Sb9, Sb29, Sb30, and Sb40 showed promising performance based on the combined interpretation of MP, GMP, and STI, indicating their potential value as genetic resources for further drought tolerance breeding efforts. The observed variation among genotypes identified through drought tolerance indices further highlights that genotype classification should not rely on a single index, but rather on the integrated assessment of yield performance and multiple drought response parameters. The GGE biplot analysis revealed differences in genotype stability across contrasting environments. Genotypes located closer to the origin exhibited lower genotype × environment interaction effects and greater yield stability across evaluated environments. Among the evaluated genotypes, Sb8, Sb30, Sb40 and Sb51 showed relatively favourable combinations of stability and positive performance, whereas genotypes positioned farther from the origin displayed greater environmental sensitivity.

Based on the combined evaluation of MP, GMP, and STI, genotypes Sb7, Sb8, Sb9, Sb29, Sb30 and Sb40 were identified as promising performers, exhibiting high yield potential and relatively stable performance across contrasting water regimes. These genotypes may represent valuable genetic resources for drought tolerance breeding programs due to their ability to maintain productivity under variable environmental conditions.

Instead, tolerance indices should be interpreted jointly with actual grain yield under both irrigated and rainfed conditions. Although associations were detected among some agronomic and biochemical traits, the present results are insufficient to classify these traits as validated biochemical or morphological markers of genotypes with improved performance under water limitation.

Overall, SHW germplasm represents a potentially valuable source of variation for improving wheat productivity and tolerance to water-limited environments. However, the present findings were obtained under the environmental conditions of a single experimental site and should therefore be interpreted cautiously. Further multilocation and multiyear evaluations are necessary to confirm the consistency of yield performance and drought tolerance among the promising synthetic wheat genotypes identified in this study before their incorporation into wheat-breeding programmes. Further physiological and molecular studies may also help clarify the mechanisms underlying their contrasting responses to water deficit.

## Figures and Tables

**Table 1:** Descriptive statistics and phenotypic variation in agronomic and grain quality traits in synthetic wheat genotypes.

Trait	Mean	SD	SE	Variance	CV (%)	Min	Max
Plant Height	116.059	19.298	0.560	372.421	16.628	67.000	170.000
Peduncle Length	40.918	6.808	0.198	46.345	16.637	4.400	67.000
Flag Leaf Length	20.786	5.055	0.147	25.548	24.317	8.780	48.000
Biomass	4.558	0.720	0.021	0.518	15.794	2.600	7.000
Unfilled grains	1.866	1.294	0.038	1.673	69.307	0.000	8.000
Thousand Kernel Weight	45.128	5.587	0.162	31.214	12.380	18.000	61.380
Spikelets per Spike	16.734	2.594	0.075	6.726	15.498	9.000	26.000
Spike length	11.123	2.018	0.059	4.072	18.142	3.600	19.300
Spike Weight	2.216	0.987	0.029	0.975	44.544	0.500	13.000
Grain protein content (%)	14.417	1.723	0.050	2.968	11.951	8.900	20.000
Lysine %	1.805	0.412	0.012	0.169	22.794	0.780	3.100
Tryptophan %	0.696	0.137	0.004	0.019	19.680	0.100	1.300
Grain yield (t/ha)	4.004	0.763	0.022	0.582	19.047	1.800	5.900

**Table 2:** Analysis of variance (F-values) for agronomic and grain-quality traits under contrasting water regimes across growing seasons.

Trait	G	W	Y	G × W	G × Y	W × Y	G × W × Y
Plant height	15.27 ***	788.75 ***	589.80 ***	14.96 ***	11.18 ***	666.56 ***	10.10 ***
Peduncle length	3.30 ***	7.94 *	16.91 ***	2.38 ***	1.82 ***	12.68 **	1.95 ***
Flag-leaf length	60.42 ***	2.10 ns	161.81 ***	62.65 ***	32.04 ***	295.29 ***	25.28 ***
Biomass	31.52 ***	2289.06 ***	317.23 ***	28.62 ***	16.85 ***	463.22 ***	12.93 ***
Unfilled grains	460.02 ***	11,706.56 ***	1549.46 ***	195.57 ***	120.39 ***	59.26 ***	134.37 ***
Thousand-kernel weight	65.80 ***	4.02 ns	10.91 **	3.52 ***	0.56 ns	23.03 ***	0.83 ns
Spikelets per spike	3.88 ***	7.76 *	50.69 ***	3.32 ***	1.85 ***	39.07 ***	1.69 ***
Spike length	1.05 ns	0.24 ns	1.39 ns	1.02 ns	1.01 ns	0.58 ns	1.00 ns
Spike weight	1.03 ns	3.32 ns	1.34 ns	1.05 ns	1.00 ns	1.43 ns	1.00 ns
Grain yield (t/ha)	7.04 ***	23.30 ***	92.39 ***	9.12 ***	5.89 ***	50.81 ***	4.34 ***
Grain protein content (%)	2.24 ***	22.30 ***	3.25 ns	1.45 *	1.01 ns	0.16 ns	1.18 ns
Lysine content (%)	5.11 ***	74.55 ***	2.29 ns	3.47 ***	1.63 ***	7.85 **	1.28 *
Tryptophan content (%)	0.98 ns	1.83 ns	0.70 ns	1.01 ns	1.00 ns	0.71 ns	1.01 ns

Note: G, genotype; W, water regime/environment (irrigated and rainfed conditions); Y, growing season. Genotype, water regime/environment, growing season, and their interactions were treated as fixed effects, whereas blocks nested within each year × water-regime combination were treated as random effects. F-tests for W, Y, and W × Y used the block-within-year × water-regime variation as the corresponding error stratum, whereas genotype and genotype-associated interactions were tested against plot-level residual variation. ns, not significant (*p* ≥ 0.05); * *p* < 0.05; ** *p* < 0.01; *** *p* < 0.001.

**Table 3:** Grain yield performance and drought tolerance indices of synthetic wheat genotypes under contrasting water regimes.

Genotypes	Yp (t ha^−1^)	Ys (t ha^−1^)	MP	GMP	TOL	SSI	STI
Sb1	4.461	2.644	3.553	3.435	1.817	−12.719	0.76
Sb 2	3.6	3.256	3.428	3.423	0.344	−2.988	0.755
Sb 3	3.006	2.533	2.769	2.759	0.472	−4.907	0.49
Sb 4	3.277	3.011	3.144	3.141	0.266	−2.531	0.635
Sb 5	4.044	4.611	4.328	4.318	−0.567	4.376	1.201
Sb 6	4.5	4.3	4.4	4.399	0.2	−1.388	1.246
Sb 7	3.9	4.778	4.339	4.317	−0.878	7.03	1.2
Sb 8	4.461	2.644	3.553	3.435	1.817	−12.719	1.25
Sb 9	3.556	3.978	3.767	3.761	−0.422	3.709	0.911
Sb 10	3.567	4.122	3.844	3.834	−0.556	4.865	0.947
Sb 11	3.611	4.567	4.089	4.061	−0.956	8.265	1.062
Sb 12	3.411	3.989	3.7	3.689	−0.578	5.29	0.876
Sb 13	3.45	4.7	4.075	4.027	−1.25	11.316	1.044
Sb 14	3.8	4.589	4.194	4.176	−0.789	6.484	1.123
Sb 15	3.7	4.589	4.144	4.121	−0.889	7.504	1.093
Sb 16	3.6	4.644	4.122	4.089	−1.044	9.062	1.077
Sb 17	3.578	4.644	4.111	4.076	−1.067	9.312	1.07
Sb 18	4.339	3.483	3.911	3.888	0.856	−6.159	0.973
Sb 19	3.944	4.128	4.036	4.035	−0.183	1.452	1.049
Sb 20	3.483	3.961	3.722	3.715	−0.478	4.284	0.889
Sb 21	4.217	4.222	4.219	4.219	−0.006	0.041	1.147
Sb 22	3.733	4.033	3.883	3.88	−0.3	2.51	0.97
Sb 23	3.761	4.067	3.914	3.911	−0.306	2.537	0.985
Sb 24	3.333	4.344	3.839	3.805	−1.011	9.474	0.933
Sb 25	4.406	4.378	4.392	4.392	0.028	−0.197	1.242
Sb 26	4.222	4.433	4.328	4.326	−0.211	1.562	1.206
Sb 27	4.633	3.756	4.194	4.171	0.878	−5.917	1.121
Sb 28	4.178	3.878	4.028	4.025	0.3	−2.243	1.043
Sb 29	3.8	4.078	3.939	3.936	−0.278	2.283	0.998
Sb 30	4.278	3.856	4.067	4.061	0.422	−3.083	1.062
Sb 31	4.044	4.167	4.106	4.105	−0.122	0.944	1.085
Sb 32	4.044	4.222	4.133	4.132	−0.178	1.373	1.1
Sb 33	3.767	4.333	4.05	4.04	−0.567	4.699	1.051
Sb 34	3.767	4.344	4.056	4.045	−0.578	4.791	1.054
Sb 35	3.911	4.456	4.183	4.174	−0.544	4.348	1.122
Sb 36	4.167	4.567	4.367	4.362	−0.4	2.998	1.225
Sb 37	3.689	4.156	3.922	3.915	−0.467	3.951	0.987
Sb 38	3.978	3.944	3.961	3.961	0.033	−0.262	1.01
Sb 39	4.133	4.267	4.2	4.199	−0.133	1.008	1.136
Sb 40	3.7	4.133	3.917	3.911	−0.433	3.658	0.985
Sb 41	3.711	4.489	4.1	4.082	−0.778	6.546	1.073
Sb 42	4.589	3.822	4.206	4.188	0.767	−5.218	1.13
Sb 43	4.3	4.044	4.172	4.17	0.256	−1.856	1.12
Sb 44	4.244	4.144	4.194	4.194	0.1	−0.736	1.133
Sb 45	4.456	4.022	4.239	4.233	0.433	−3.038	1.154
Sb 46	4.033	4.044	4.039	4.039	−0.011	0.086	1.051
Sb 47	3.633	4.311	3.972	3.958	−0.678	5.826	1.009
Sb 48	3.822	4.167	3.994	3.991	−0.344	2.815	1.026
Sb 49	3.622	4.456	4.039	4.017	−0.833	7.186	1.039
Sb 50	4.011	4.433	4.222	4.217	−0.422	3.288	1.145
Sb 51	3.467	4.148	3.807	3.792	−0.681	6.137	0.926
Sb 52	3.656	4.467	4.061	4.041	−0.811	6.93	1.052
Sb 53	3.889	3.792	3.841	3.84	0.097	−0.776	0.95
Sb 54	3.667	4.033	3.85	3.846	−0.367	3.123	0.952
Sb 55	4.4	4.333	4.367	4.367	0.067	−0.473	1.228
Sb 56	4.067	3.822	3.944	3.943	0.244	−1.877	1.001
Sb 57	4.544	3.622	4.083	4.057	0.922	−6.338	1.06
Sb 58	4.511	4.011	4.261	4.254	0.5	−3.462	1.165
Sb 59	4.389	3.467	3.928	3.901	0.922	−6.563	0.98
Sb 60	4.114	3.656	3.885	3.878	0.459	−3.483	0.969
Sb 61	4.048	4.033	4.041	4.041	0.014	−0.111	1.051
Sb 62	3.878	3.759	3.818	3.818	0.119	−0.958	0.939
Sb 63	4.467	3.833	4.15	4.138	0.633	−4.429	1.103
Sb 64	3.714	4.014	3.864	3.862	−0.3	2.523	0.96
Sb 65	3.933	4.344	4.139	4.134	−0.411	3.265	1.101
Sb 66	3.856	4.322	4.089	4.082	−0.467	3.78	1.073

Yp—yield under irrigated conditions; Ys—yield under rainfed conditions; MP—mean productivity; GMP—geometric mean productivity; TOL—tolerance index; SSI—stress susceptibility index; STI—stress tolerance index.

**Table 4:** Correlation coefficients between Yp, Ys and the indices of synthetic wheat.

	Yp	Ys	TOL	MP	GMP	SSI	STI
Yp	1	−0.137	0.679 **	0.544 **	0.524 **	−0.668 **	0.531 **
Ys	−0.137	1	−0.820 **	0.757 **	0.768 **	0.823 **	0.759 **
TOL	0.679 **	−0.820 **	1	−0.247	−0.267	−0.995 **	−0.256
MP	0.544 **	0.757 **	−0.247	1	0.997 **	0.256	0.994 **
GMP	0.524 **	0.768 **	−0.267	0.997 **	1	0.270	0.997 **
SSI	−0.668 **	0.823 **	−0.995 **	0.256	0.270	1	0.256
STI	0.531 **	0.759 **	−0.256	0.994 **	0.997 **	0.256	1

Note: ** *p* < 0.01.

**Table 5:** Principal component analysis loading values and variance contribution of agronomic and grain-quality traits in synthetic wheat genotypes.

Trait	PC1	PC2	PC3	PC4	PC5
Plant Height	0.636	−0.152	−0.007	−0.045	0.050
Peduncle Length	0.153	−0.420	0.118	0.141	−0.456
Flag Leaf Length	0.165	−0.459	0.039	0.184	−0.218
Biomass	0.586	0.026	0.039	−0.271	0.280
Unfilled grains	0.379	0.528	0.164	0.165	0.031
Thousand-kernel weight	0.102	−0.010	0.159	0.763	0.176
Spikelets per spike	0.032	−0.491	−0.390	−0.051	0.455
Spike length	0.004	−0.051	−0.005	0.348	−0.008
Spike weight	−0.132	−0.005	0.003	0.288	0.502
Grain protein content (%)	0.148	0.206	−0.476	0.201	−0.393
Lysine content (%)	−0.054	−0.151	0.742	−0.112	0.018
Tryptophan content (%)	−0.065	0.025	−0.040	0.038	0.131

**Table 6:** Pedigree information and genetic background of SHW genotypes.

N	Synthetic ID	Genotype Code	Pedigree/Genetic Line
	CIMMYT lines		
1	Sb1	S-YT-4	AISBERG/AE.SQUARROSA (511)
2	Sb2	S-E-50	AISBERG/AE.SQUARROSA (369)
3	Sb3	S-YT-6	UKR-OD 1530.94/AE.SQUARROSA (310)
4	Sb4	S-YT-8	UKR-OD 952.92/AE.SQUARROSA (1031)
5	Sb5	S-YT-9	UKR-OD 1530.94/AE.SQUARROSA (458)
6	Sb6	S-YT-10	UKR-OD 1530.94/AE.SQUARROSA (458)
	CYMMIT winter synthetics × modern varieties		
7	Sb7	S-YT-11	AISBERG/AE.SQUARROSA (369)/DEMIR
8	Sb8	S-YT-12	AISBERG/AE.SQUARROSA (369)/DEMIR
9	Sb9	S-YT-13	LEUC 84693/AE.SQUARROSA (310)/ADYR
10	Sb10	S-YT-15	LEUC 84693/AE.SQUARROSA (310)/ADYR
11	Sb11	S-YT-16	LEUC 84693/AE.SQUARROSA (1026)/GEREK79
12	Sb12	S-YT-17	UKR-OD 952.92/AE.SQUARROSA (409)/SONMEZ
13	Sb13	S-YT-18	UKR-OD 952.92/AE.SQUARROSA (409)/SONMEZ
14	Sb14	S-YT-19	UKR-OD 1530.94/AE.SQUARROSA (446)/KATIA1
15	Sb15	S-YT-21	UKR-OD 1530.94/AE.SQUARROSA (311)/EKIZ
16	Sb16	S-YT-22	UKR-OD 1530.94/AE.SQUARROSA (311)/EKIZ
17	Sb17	S-YT-23	UKR-OD 1530.94/AE.SQUARROSA (311)/EKIZ
18	Sb18	S-YT-24	UKR-OD 1530.94/AE.SQUARROSA (312)/BAGCI2002
19	Sb19	S-YT-25	UKR-OD 1530.94/AE.SQUARROSA (312)/BAGCI2002
20	Sb20	S-E-26	LEUC 84693/AE.SQUARROSA (1026)/GEREK79
21	Sb21	S-E-27	UKR-OD 1871.94/AE.SQUARROSA (213)/MEZGIT-6
22	Sb22	S-E-28	UKR-OD 952.92/AE.SQUARROSA (409)/SONMEZ
23	Sb23	S-E-29	UKR-OD 952.92/AE.SQUARROSA (409)/SONMEZ
24	Sb24	S-E-31	UKR-OD 952.92/AE.SQUARROSA(409)/SONMEZ
25	Sb25	S-E-32	UKR-OD 952.92/AE.SQUARROSA (409)/SONMEZ
26	Sb26	S-E-33	UKR-OD 1530.94/AE.SQUARROSA (446)/KATIA1
27	Sb27	S-E-34	UKR-OD 1530.94/AE.SQUARROSA (446)/KATIA1
28	Sb28	S-E-35	UKR-OD 1530.94/AE.SQUARROSA (446)/KATIA1
29	Sb29	S-E-36	UKR-OD 1530.94/AE.SQUARROSA (446)/KATIA1
30	Sb30	S-E-37	UKR-OD 1530.94/AE.SQUARROSA (446)/KATIA1
31	Sb31	S-E-38	UKR-OD 1530.94/AE.SQUARROSA (311)/EKIZ
32	Sb32	S-E-39	UKR-OD 1530.94/AE.SQUARROSA (311)/EKIZ
33	Sb33	S-E-41	UKR-OD 1530.94/AE.SQUARROSA (311)/EKIZ
34	Sb34	S-E-42	UKR-OD 1530.94/AE.SQUARROSA (311)/EKIZ
35	Sb35	S-E-44	UKR-OD 1530.94/AE.SQUARROSA (312)/BAGCI2002
36	Sb36	S-E-45	UKR-OD 1530.94/AE.SQUARROSA (312)/BAGCI2002
37	Sb37	S-E-46	UKR-OD 1530.94/AE.SQUARROSA (312)/BAGCI2002
38	Sb38	S-E-47	UKR-OD 1530.94/AE.SQUARROSA (312)/BAGCI2002
39	Sb39	S-E-48	UKR-OD 1530.94/AE.SQUARROSA (312)/BAGCI2002
40	Sb40	S-E-49	AISBERG/AE.SQUARROSA (369)
41	Sb41	S-E-51	LEUC 84693/AE.SQUARROSA (1026)
42	Sb42	S-E-53	UKR-OD 1530.94/AE.SQUARROSA (310)
43	Sb43	S-E-55	UKR-OD 1530.94/AE.SQUARROSA (310)
44	Sb44	S-E-56	UKR-OD 1530.94/AE.SQUARROSA (458)
45	Sb45	S-E-57	UKR-OD 1530.94/AE.SQUARROSA (629)
46	Sb46	S-E-58	UKR-OD 1530.94/AE.SQUARROSA (629)
47	Sb47	S-E-61	AISBERG/AE.SQUARROSA (369)/DEMIR
48	Sb48	S-E-62	AISBERG/AE.SQUARROSA (369)/DEMIR
49	Sb49	S-E-63	LEUC 84693/AE.SQUARROSA (310)/ADYR
50	Sb50	S-E-64	LEUC 84693/AE.SQUARROSA (1026)/GEREK79
51	Sb51	S-E-65	UKR-OD 1871.94/AE.SQUARROSA (213)/MEZGIT-6
52	Sb52	S-E-66	UKR-OD 1871.94/AE.SQUARROSA (213)/MEZGIT-6
53	Sb53	S-E-67	UKR-OD 952.92/AE.SQUARROSA (409)/SONMEZ
	Synthetic lines of the University of Kyoto		
54	Sb54	J-3	LANGDON/AE 929
55	Sb55	J-4	LANGDON/IG 48042
56	Sb56	J-5	LANGDON/IG 126387
57	Sb57	J-12	LANGDON/KU-2092
58	Sb58	J-15	LANGDON/KU-2098
59	Sb59	J-16	LANGDON/KU-2100
60	Sb60	J-17	LANGDON/KU-2105
61	Sb61	J-18	LANGDON/KU-2159
62	Sb62	J-19	LANGDON/KU-2829A
63	Sb63	J-25	LANGDON/KU-20-10
64	Sb64	J-26	LANGDON/KU-2078
65	Sb65	J-27	LANGDON/KU-2079
66	Sb66	J-35	LANGDON/PI 499262

Note: KU—Plant Germ-Plasm Institute, Faculty of Agriculture, Kyoto University, IG—International Center for Agricultural Research in the Dry Areas (ICARDA).

## Data Availability

The data presented in this study are available within the article and its Supplementary Materials.

## Supplementary Materials

The following supporting information can be downloaded at: https://www.mdpi.com/article/10.3390/plants15193054/s1, Table S1: Mean comparison of agronomic and grain-quality traits among synthetic wheat genotypes based on Tukey’s HSD test; Table S2: Summary of selected synthetic wheat genotypes based on drought tolerance indices; Table S3: Meteorological data for the 2022–2025 Growing Season at the Absheron Scientific Research Base Experimental Station of the Institute of Genetic Resources; Table S4: Irrigation schedule applied in the irrigated treatment during the winter wheat growing seasons; Table S5: Soil water content (θ) and field capacity (FC) across the growing season.

## References

[1] United Nations Convention to Combat Desertification (UNCCD) (2024). The Global Threat of Drying Lands: Regional and Global Aridity Trends and Future Projections.

[2] United Nations Convention to Combat Desertification (UNCCD) (2024). COP16: Two-Month Media Primer.

[3] Ahmed M., Tóth Z., Decsi K. (2024). The impact of salinity on crop yields and the confrontational behavior of transcriptional regulators, nanoparticles, and antioxidant defensive mechanisms under stressful conditions: A review. Int. J. Mol. Sci..

[4] Muhammad M., Waheed A., Wahab A., Majeed M., Nazim M., Liu Y.H., Li L., Li W.J. (2024). Soil salinity and genotypes with improved performance under water limitation: An evaluation of plant growth, productivity, microbial diversity, and amelioration strategies. Plant Stress.

[5] Haghpanah M., Hashemipetroudi S., Arzani A., Araniti F. (2024). Genotypes with improved performance under water limitation in plants: Physiological and molecular responses. Plants.

[6] Nyaupane S., Sharma R.C., Shrestha S., Bhattarai S. (2024). Drought stress effect, tolerance, and management in wheat. Cogent Food Agric..

[7] Erenstein O., Jaleta M., Mottaleb K.A., Sonder K., Donovan J., Braun H.J., Reynolds M.P., Braun H.J. (2022). Global trends in wheat production, consumption and trade. Wheat Improvement.

[8] Mourad A.M.I., Morgounov A., Baenziger P.S., Esmail S.M. (2022). Genetic Variation in Common Bunt Resistance in Synthetic Hexaploid Wheat. Plants.

[9] Wan H., Yang F., Li J., Wang Q., Liu Z., Tang Y., Yang W. (2023). Genetic improvement and application practices of synthetic hexaploid wheat. Genes.

[10] Liu Z., Yang F., Deng C., Wan H., Tang H., Feng J., Wang Q., Yang N., Li J., Yang W. (2024). Chromosome-level assembly of the synthetic hexaploid wheat-derived cultivar Chuanmai 104. Sci. Data.

[11] Wan H., Li J., Ma S., Yang F., Chai L., Liu Z., Wang Q., Pu Z., Yang W. (2022). Allopolyploidization increases genetic recombination in the ancestral diploid D genome during wheat evolution. Crop J..

[12] Mokhtari N., Majidi M.M., Mirlohi A. (2025). Synthetic wheat as a new source of flour quality under drought conditions: Associations with solvent retention capacity. PLoS ONE.

[13] Song Q., Cui W., Song J., Zhou B., Chen L., Xu K., Jin Y. (2026). Uncover the genetic basis of processing quality related traits in common wheat (*Triticum aestivum* L.) using genome-wide association mapping. Front. Plant Sci..

[14] Khalid A., Hameed A., Tahir M.F. (2023). Wheat quality: A review on chemical composition, nutritional attributes, grain anatomy, types, classification, and function of seed storage proteins in bread making quality. Front. Nutr..

[15] Andrade J.F., Man J., Monzon J.P., Rattalino Edreira J.I., Yuan S., Lollato R.P., de la Vega A.J., Llorens C., Silva A.d.O., Peng S. (2026). Maintenance breeding and breeding for yield potential both contribute to genetic improvement in wheat yield. Nat. Commun..

[16] Ahmed K., Shabbir G., Ahmed M., Shah K.N. (2020). Phenotyping for drought resistance in bread wheat using physiological and biochemical traits. Sci. Total Environ..

[17] Rharrabti Y., Elhani S., Martos-Núñez V., García Del Moral L.F. (2001). Protein and lysine content, grain yield, and other technological traits in durum wheat under Mediterranean conditions. J. Agric. Food Chem..

[18] Yadav A.K., Carroll A.J., Estavillo G.M., Rebetzke G.J., Pogson B.J. (2019). Wheat genotypes with improved performance under water limitation in the field is predicted by amino acid responses to glasshouse-imposed drought. J. Exp. Bot..

[19] Yu M., Liu Z.H., Yang B., Chen H., Zhang H., Hou D.B. (2020). The contribution of photosynthesis traits and plant height components to plant height in wheat at the individual quantitative trait locus level. Sci. Rep..

[20] Isgandarova T.Y., Rustamova S.M., Aliyeva D.R., Rzayev F.H., Gasimov E.K., Huseynova I.M. (2024). Antioxidant and Ultrastructural Alterations in Wheat During Drought-Induced Leaf Senescence. Agronomy.

[21] Wang X., Huang J., Peng S., Xiong D. (2023). Leaf rolling precedes stomatal closure in rice (*Oryza sativa*) under drought conditions. J. Exp. Bot..

[22] Gurbanova U.A., Aliyeva D.R., Jahangirov A.A., Isgandarova T.Y., Zulfugarova S.T., Huseynova I.M. (2026). Integrated Assessment of Morphophysiological and Biochemical Traits for Identifying Drought-Tolerant Bread Wheat Genotypes Under Rainfed Conditions. Agronomy.

[23] Singh C., Yadav S., Khare V., Gupta V., Patial M., Kumar S., Mishra C.N., Tyagi B.S., Gupta A., Sharma A.K. (2025). Wheat genotypes with improved performance under water limitation: Unveiling a synergistic future with conventional and molecular breeding strategies. Plants.

[24] Rezaei V., Majidi M.M., Mirlohi A., Saeidi G. (2026). Productivity and quality-related traits of wheat germplasm affected by heat stress. PLoS ONE.

[25] Bhandari R., Paudel H., Alharbi S.A., Ansari M.J., Poudel M.R., Neupane M.P., Solanki P. (2024). Evaluating stress tolerance indices for their comparative validity to assess heat and genotypes with improved performance under water limitation of winter wheat genotypes. J. Agric. Food Res..

[26] Zulfugarova S.T., Rustamova S.M., Pashayeva A.N., Rzayev F.H., Gasimov E.K., Huseynova I.M. (2026). Integrated Assessment of Physiological, Molecular and Ultrastructural Responses to Heat Stress in Wheat. Plants.

[27] Pashayeva A.N. (2026). STN7/STN8-Mediated Thylakoid Protein Phosphorylation: From Redox Signaling to Photosynthetic Acclimation Under Abiotic Stress. BioChem.

[28] Tadesse W., Sanchez-Garcia M., Gizaw S., Amri A., Bishaw Z., Ogbonnaya F.C., Baum M. (2019). Genetic gains in wheat breeding and its role in feeding the world. Crop Breed. Genet. Genom..

[29] Vinklárková B. (2015). The Kjeldahl method as a primary reference procedure for total protein in certified reference materials used in clinical chemistry. Crit. Rev. Anal. Chem..

[30] Museyko A.S., Sysoev A.F. (1970). Determination of lysine in seeds. Rep. VASKhNIL.

[31] Ermakov A.I., Yarosh N.P., Mikhailov A.A. (1969). Determination of tryptophan in seeds. Bull. VIR.

[32] Ali M.B., El Sadek A.N. (2016). Evaluation of genotypes with improved performance under drought tolerance indices for wheat (*Triticum aestivum* L.) under irrigated and rainfed conditions. Commun. Biometry Crop Sci..

[33] Rijal R.B., Bashyal P., Yadav D.R., Upadhyay K., Pant K.R., Adhikari N.R. (2024). Assessing genotypes with improved performance under water limitation in advanced wheat genotypes using stress tolerance indices. Arch. Agric. Environ. Sci..

