# Supplementary material for: Multi-Year Characterisation of Drought Adaptation and Grain Quality in Synthetic Hexaploid Wheat Genotypes
Source: Plants (Basel). 2026 Oct 7;15(19):3054. doi: 10.3390/plants15193054 (PMC13666747; doi:10.3390/plants15193054)
Supplement: Supplementary file 1 [file plants-15-03054-s001.zip › Supplementary Table S1.pdf]

Table S1. Mean comparison of agronomic and grain-quality traits among synthetic wheat genotypes based on Tukey's HSD test.

Parameter	Plant Height	Peduncle Length	Flag Leaf Length	Biomass	Spike length	Spike Weight	Spikelets per Spike	Thousand Kernel Weight	Grain yield (t/ha)	Grain protein content (%)	Lysine (%)	Tryptophan(%)
Sb1	115.27 ± 5.32a	35.02 ± 1.71b	23.62 ± 0.46a	4.46 ± 0.18a	10.13 ± 0.18b	2.53 ± 0.08a	14.07 ± 0.60b	52.56 ± 0.57a	3.55 ± 0.24a	14.84 ± 0.25a	1.68 ± 0.06b	0.75 ± 0.04a
Sb2	122.38 ± 5.29a	40.90 ± 2.61b	23.41 ± 0.80a	4.89 ± 0.25a	10.36 ± 0.35b	1.60 ± 0.20b	15.73 ± 0.76b	47.11 ± 0.40b	3.43 ± 0.23a	15.10 ± 0.43a	1.95 ± 0.14b	0.65 ± 0.02b
Sb3	117.57 ± 3.77a	41.18 ± 2.04b	22.11 ± 1.06a	4.64 ± 0.14a	10.53 ± 0.35b	2.47 ± 0.17a	16.64 ± 0.32a	44.22 ± 0.56c	2.77 ± 0.16b	15.55 ± 0.28a	1.78 ± 0.06b	0.65 ± 0.02b
Sb4	117.82 ± 3.79a	39.91 ± 1.30b	16.31 ± 0.22b	4.71 ± 0.10a	11.03 ± 0.32b	2.24 ± 0.14a	15.53 ± 0.60b	49.37 ± 0.40b	3.14 ± 0.18b	15.10 ± 0.37a	1.77 ± 0.12b	0.68 ± 0.02b
Sb5	125.07 ± 4.67a	49.59 ± 1.19a	21.65 ± 0.89a	4.80 ± 0.08a	11.37 ± 0.72b	2.09 ± 0.17a	17.56 ± 0.39a	41.66 ± 0.25d	4.33 ± 0.14a	14.82 ± 0.25a	1.58 ± 0.13b	0.63 ± 0.02b
Sb6	122.41 ± 2.34a	50.71 ± 2.33a	22.05 ± 0.91a	4.73 ± 0.10a	11.72 ± 0.56a	2.08 ± 0.24a	17.94 ± 0.49a	40.88 ± 0.40d	4.40 ± 0.08a	14.80 ± 0.26a	1.65 ± 0.04b	0.69 ± 0.01b
Sb7	125.19 ± 3.80a	43.93 ± 0.78a	21.23 ± 0.48a	4.60 ± 0.14a	10.43 ± 0.32b	2.56 ± 0.17a	17.09 ± 0.67a	45.59 ± 0.41c	4.34 ± 0.20a	14.88 ± 0.41a	1.66 ± 0.13b	0.61 ± 0.06b
Sb8	109.62 ± 3.66a	40.12 ± 1.07b	22.64 ± 0.93a	4.41 ± 0.11a	11.12 ± 0.32b	2.64 ± 0.35a	16.88 ± 0.42a	47.74 ± 0.30b	3.55 ± 0.24a	14.02 ± 0.47b	1.71 ± 0.08b	0.66 ± 0.05b
Sb9	122.56 ± 4.98a	40.80 ± 1.05b	22.59 ± 1.09a	4.19 ± 0.19a	12.41 ± 0.62a	2.11 ± 0.30a	16.83 ± 0.66a	47.27 ± 0.33b	3.77 ± 0.24a	13.99 ± 0.34b	1.65 ± 0.08b	0.66 ± 0.02b
Sb10	115.84 ± 5.05a	41.39 ± 1.05b	20.58 ± 0.45a	4.97 ± 0.23a	9.85 ± 0.32b	2.48 ± 0.21a	16.79 ± 0.51a	40.65 ± 0.35d	3.84 ± 0.10a	15.59 ± 0.33a	1.68 ± 0.08b	0.65 ± 0.02b
Sb11	113.33 ± 5.39a	40.32 ± 1.17b	23.52 ± 0.92a	4.26 ± 0.22a	10.67 ± 0.30b	2.30 ± 0.18a	17.03 ± 0.74a	42.13 ± 0.93d	4.09 ± 0.14a	12.66 ± 0.45b	1.97 ± 0.12b	0.86 ± 0.03a
Sb12	103.53 ± 5.79a	35.17 ± 1.19b	17.09 ± 0.78b	4.67 ± 0.11a	9.68 ± 0.26b	1.97 ± 0.13a	17.02 ± 0.39a	47.46 ± 0.43b	3.70 ± 0.19a	13.00 ± 0.23b	2.04 ± 0.12a	0.73 ± 0.03a
Sb13	110.06 ± 4.59a	37.35 ± 1.25b	20.05 ± 1.09a	4.76 ± 0.15a	10.80 ± 0.49b	2.16 ± 0.19a	17.15 ± 0.52a	46.35 ± 0.48b	4.11 ± 0.18a	14.04 ± 0.35b	1.93 ± 0.10b	0.70 ± 0.02a
Sb14	117.54 ± 5.12a	38.19 ± 1.32b	19.18 ± 0.48a	4.60 ± 0.21a	10.17 ± 0.35b	2.57 ± 0.15a	16.08 ± 0.74a	39.46 ± 0.58d	4.19 ± 0.11a	16.14 ± 0.25a	1.68 ± 0.07b	0.64 ± 0.01b
Sb15	117.69 ± 3.72d	40.86 ± 0.64b	19.45 ± 0.54a	4.93 ± 0.17a	12.07 ± 0.31a	2.53 ± 0.21a	18.03 ± 0.48a	45.15 ± 0.49c	4.14 ± 0.12a	14.64 ± 0.23a	1.68 ± 0.05b	0.64 ± 0.02b
Sb16	115.67 ± 2.38a	39.46 ± 0.60b	19.68 ± 1.36a	4.80 ± 0.09a	12.25 ± 0.38a	2.59 ± 0.13a	17.20 ± 0.46a	45.53 ± 0.27c	4.12 ± 0.13a	14.73 ± 0.32a	1.77 ± 0.06b	0.65 ± 0.02b
Sb17	118.30 ± 4.30a	38.43 ± 0.82b	24.47 ± 1.23a	4.87 ± 0.18a	11.57 ± 0.31a	2.73 ± 0.20a	18.13 ± 0.65a	53.73 ± 0.81a	4.11 ± 0.14a	13.80 ± 0.32b	1.81 ± 0.11b	0.73 ± 0.02a
Sb18	112.63 ± 4.41a	39.01 ± 1.66b	25.03 ± 1.82a	4.86 ± 0.21a	10.90 ± 0.52b	2.70 ± 0.24a	16.42 ± 0.66a	40.98 ± 0.67d	3.91 ± 0.18a	14.35 ± 0.32a	1.89 ± 0.04b	0.71 ± 0.03a
Sb19	112.68 ± 2.73a	43.97 ± 0.91a	22.78 ± 1.09a	3.93 ± 0.05b	9.83 ± 0.40b	2.29 ± 0.19a	16.03 ± 0.45b	52.90 ± 0.54a	4.04 ± 0.16a	14.09 ± 0.36a	1.71 ± 0.07b	0.71 ± 0.04a
Sb20	103.15 ± 3.15a	38.36 ± 1.78b	21.38 ± 0.79a	3.98 ± 0.14b	9.02 ± 0.30c	1.80 ± 0.18b	16.22 ± 0.53a	40.68 ± 0.33d	3.72 ± 0.14a	14.61 ± 0.25a	1.39 ± 0.09c	0.73 ± 0.03a
Sb21	109.76 ± 2.96c	39.90 ± 1.25b	20.71 ± 0.63a	4.36 ± 0.12a	9.65 ± 0.23b	1.97 ± 0.13a	16.35 ± 0.75a	44.29 ± 0.65c	4.22 ± 0.15a	14.45 ± 0.39a	1.58 ± 0.09b	0.75 ± 0.05a
Sb22	107.27 ± 4.11a	38.75 ± 0.69b	18.62 ± 0.59b	4.21 ± 0.15a	10.14 ± 0.46b	2.83 ± 0.43a	15.82 ± 0.55b	41.42 ± 0.36d	3.88 ± 0.14a	14.69 ± 0.40a	1.45 ± 0.13c	0.66 ± 0.02b
Sb23	111.87 ± 4.28a	40.68 ± 1.25b	21.38 ± 0.77a	4.39 ± 0.16a	10.70 ± 0.29b	2.25 ± 0.17a	16.40 ± 0.64a	45.31 ± 0.67c	3.91 ± 0.14a	14.74 ± 0.40a	1.56 ± 0.15b	0.72 ± 0.02a
Sb24	114.41 ± 5.19a	39.95 ± 1.37b	21.64 ± 0.90a	4.07 ± 0.12b	11.01 ± 0.40b	2.02 ± 0.19a	18.14 ± 0.68a	42.12 ± 0.45d	3.84 ± 0.15a	13.92 ± 0.27b	1.37 ± 0.09c	0.71 ± 0.03a
Sb25	115.97 ± 5.03a	41.32 ± 0.77b	23.11 ± 0.52a	4.47 ± 0.19a	10.34 ± 0.32b	2.30 ± 0.28a	17.47 ± 0.52a	41.48 ± 0.74d	4.39 ± 0.14a	14.20 ± 0.54a	1.46 ± 0.11c	0.66 ± 0.03b
Sb26	114.74 ± 3.35a	37.82 ± 1.36b	20.74 ± 0.96a	4.17 ± 0.09a	10.75 ± 0.40b	2.96 ± 0.16a	18.27 ± 0.60a	40.88 ± 0.53d	4.33 ± 0.18a	14.66 ± 0.45a	1.75 ± 0.14b	0.65 ± 0.04b
Sb27	124.76 ± 4.70a	42.31 ± 1.22a	17.86 ± 0.59b	5.00 ± 0.14a	11.55 ± 0.47b	2.95 ± 0.64a	17.40 ± 0.83a	41.84 ± 0.70d	4.19 ± 0.23a	14.94 ± 0.20a	1.68 ± 0.08b	0.59 ± 0.03b
Sb28	117.21 ± 3.07a	38.54 ± 1.23b	19.56 ± 0.70a	5.03 ± 0.19a	10.81 ± 0.45b	2.64 ± 0.10a	15.70 ± 0.48b	39.00 ± 1.11d	4.03 ± 0.23a	10.66 ± 0.27c	2.28 ± 0.10a	0.86 ± 0.07a
Sb29	111.55 ± 3.41a	37.16 ± 1.43b	19.00 ± 1.19a	4.47 ± 0.13a	11.46 ± 0.46b	2.29 ± 0.14a	16.88 ± 0.42a	42.32 ± 0.97d	3.94 ± 0.21a	13.49 ± 0.26b	1.88 ± 0.03b	0.61 ± 0.02b
Sb30	116.21 ± 4.00a	38.98 ± 0.94b	23.18 ± 0.89a	4.78 ± 0.09a	12.67 ± 0.28a	2.57 ± 0.21a	18.80 ± 0.51a	45.37 ± 0.49c	4.07 ± 0.16a	13.80 ± 0.35b	2.08 ± 0.08a	0.77 ± 0.02a
Sb31	110.29 ± 3.74a	40.01 ± 1.34b	25.20 ± 1.73a	4.87 ± 0.06a	12.52 ± 0.45a	3.06 ± 0.28a	18.07 ± 0.55a	45.49 ± 0.53c	4.11 ± 0.14a	15.17 ± 0.26a	1.51 ± 0.08c	0.67 ± 0.03b
Sb32	118.88 ± 3.18a	39.42 ± 0.43b	23.20 ± 1.32a	4.91 ± 0.07a	11.89 ± 0.62a	2.06 ± 0.16a	17.46 ± 0.72a	42.56 ± 0.54c	4.13 ± 0.10a	15.08 ± 0.26a	1.68 ± 0.09b	0.75 ± 0.02a
Sb33	117.04 ± 3.99a	36.32 ± 1.03b	22.52 ± 1.11a	4.78 ± 0.13a	12.03 ± 0.34a	3.09 ± 0.16a	18.98 ± 0.63a	53.49 ± 0.72a	4.05 ± 0.16a	13.26 ± 0.31b	1.53 ± 0.04c	0.83 ± 0.02a

Sb34	112.34 ± 3.68a	41.22 ± 1.31b	21.02 ± 0.75a	4.29 ± 0.14a	10.15 ± 0.54b	2.74 ± 0.30a	16.55 ± 0.64a	53.00 ± 0.86a	4.06 ± 0.16a	14.50 ± 0.23a	1.28 ± 0.09c	0.70 ± 0.03a
Sb35	114.99 ± 4.71a	43.51 ± 1.05a	19.28 ± 0.75a	3.98 ± 0.13b	10.60 ± 0.30b	2.65 ± 0.19a	15.54 ± 0.75b	44.10 ± 0.57c	4.18 ± 0.14a	15.12 ± 0.30a	1.61 ± 0.04b	0.71 ± 0.02a
Sb36	116.17 ± 3.02a	38.47 ± 1.04b	17.51 ± 0.63b	4.67 ± 0.13a	10.21 ± 0.25b	2.53 ± 0.12a	17.24 ± 0.44a	43.44 ± 0.68c	4.37 ± 0.15a	13.89 ± 0.31b	1.71 ± 0.06b	0.66 ± 0.04b
Sb37	111.76 ± 4.06a	37.02 ± 1.62b	19.18 ± 0.94a	4.60 ± 0.14a	10.69 ± 0.43b	1.84 ± 0.24b	16.38 ± 0.56a	44.42 ± 0.79c	3.92 ± 0.22a	15.97 ± 0.32a	1.45 ± 0.05c	0.54 ± 0.02b
Sb38	121.23 ± 3.80a	39.33 ± 0.77b	21.17 ± 0.96a	4.62 ± 0.13a	10.70 ± 0.37b	2.20 ± 0.20a	16.45 ± 0.44a	40.00 ± 0.63d	3.96 ± 0.16a	13.38 ± 0.31b	1.76 ± 0.08b	0.81 ± 0.03a
Sb39	117.47 ± 4.87a	42.71 ± 0.76a	16.91 ± 0.67b	4.67 ± 0.11a	11.04 ± 0.27b	2.67 ± 0.13a	14.39 ± 0.51b	44.72 ± 0.68c	4.20 ± 0.20a	13.57 ± 0.46b	1.59 ± 0.04b	0.67 ± 0.04b
Sb40	109.64 ± 5.58a	42.81 ± 1.35a	21.26 ± 1.37a	4.72 ± 0.21a	12.54 ± 0.33a	2.71 ± 0.28a	17.07 ± 0.64a	43.78 ± 0.64c	3.92 ± 0.21a	15.06 ± 0.34a	1.67 ± 0.04b	0.65 ± 0.02b
Sb41	117.17 ± 5.59a	45.75 ± 1.38a	18.33 ± 1.35b	4.46 ± 0.13a	11.69 ± 0.46a	1.86 ± 0.09a	16.14 ± 0.44a	45.39 ± 0.67c	4.10 ± 0.24a	12.78 ± 0.40b	2.06 ± 0.04a	0.78 ± 0.04a
Sb42	123.40 ± 5.02a	41.54 ± 1.77b	20.35 ± 1.63a	4.46 ± 0.13a	12.03 ± 0.56a	1.86 ± 0.18a	18.55 ± 0.63a	38.35 ± 0.62e	4.21 ± 0.25a	14.36 ± 0.33a	1.76 ± 0.03b	0.64 ± 0.02b
Sb43	115.37 ± 6.17a	39.99 ± 1.33b	18.20 ± 0.95b	4.61 ± 0.20a	10.03 ± 0.24b	1.39 ± 0.12b	16.33 ± 0.51a	40.34 ± 0.41d	4.17 ± 0.18a	14.26 ± 0.19a	1.81 ± 0.11b	0.69 ± 0.01b
Sb44	122.08 ± 3.40a	40.91 ± 1.48b	19.38 ± 0.89a	4.35 ± 0.14a	12.62 ± 0.49a	2.47 ± 0.27a	17.17 ± 0.43a	47.51 ± 0.79b	4.19 ± 0.16a	15.34 ± 0.35a	2.04 ± 0.04a	0.63 ± 0.02b
Sb45	114.06 ± 4.03a	42.18 ± 1.13a	21.57 ± 1.21a	4.53 ± 0.17a	12.52 ± 1.03a	2.78 ± 0.17a	16.51 ± 0.68a	50.94 ± 0.89a	4.24 ± 0.13a	13.92 ± 0.38b	2.10 ± 0.06a	0.80 ± 0.05a
Sb46	119.96 ± 4.13a	43.02 ± 0.68a	22.79 ± 1.67a	4.51 ± 0.12a	10.52 ± 0.47b	2.72 ± 0.12a	16.60 ± 0.52a	49.27 ± 0.74b	4.04 ± 0.12a	13.93 ± 0.48b	2.01 ± 0.03b	0.63 ± 0.02b
Sb47	112.76 ± 3.60a	38.20 ± 1.46b	20.16 ± 1.14a	4.19 ± 0.11a	10.40 ± 0.42b	2.09 ± 0.19a	16.03 ± 0.55b	47.03 ± 0.93b	3.97 ± 0.17a	15.10 ± 0.51a	2.04 ± 0.06a	0.71 ± 0.03a
Sb48	110.41 ± 3.97a	41.81 ± 1.23b	21.46 ± 1.09a	4.17 ± 0.09a	9.90 ± 0.37b	2.42 ± 0.03a	15.24 ± 0.58b	50.88 ± 0.76a	3.99 ± 0.14a	13.37 ± 0.39b	2.25 ± 0.09a	0.74 ± 0.04a
Sb49	112.17 ± 5.07a	40.94 ± 1.53b	24.13 ± 1.32a	4.33 ± 0.17a	10.23 ± 0.78b	1.64 ± 0.24b	14.81 ± 1.18b	39.93 ± 0.68d	4.04 ± 0.15a	12.36 ± 0.42b	2.50 ± 0.12a	0.77 ± 0.03a
Sb50	119.26 ± 5.70a	45.23 ± 1.38a	22.97 ± 0.62a	4.15 ± 0.16a	8.84 ± 0.31c	2.24 ± 0.16a	15.22 ± 0.46b	48.01 ± 0.57b	4.22 ± 0.19a	15.01 ± 0.37a	2.04 ± 0.07a	0.61 ± 0.04b
Sb51	112.47 ± 4.30a	40.86 ± 0.96b	20.40 ± 0.93a	4.55 ± 0.16a	10.69 ± 0.33b	1.96 ± 0.11a	16.67 ± 0.59a	40.70 ± 0.57d	3.81 ± 0.17a	14.60 ± 0.32a	1.71 ± 0.05b	0.64 ± 0.03b
Sb52	107.41 ± 5.39a	32.03 ± 2.15c	20.59 ± 0.84a	4.35 ± 0.17a	9.62 ± 0.40b	1.95 ± 0.22a	16.78 ± 0.47a	48.86 ± 0.95b	4.06 ± 0.20a	15.86 ± 0.23a	1.91 ± 0.04b	0.62 ± 0.02b
Sb53	113.43 ± 4.65a	39.59 ± 1.44b	24.30 ± 2.12a	4.47 ± 0.20a	11.19 ± 0.34b	1.68 ± 0.21b	17.58 ± 0.40a	45.20 ± 0.57c	3.84 ± 0.16a	13.83 ± 0.30b	1.93 ± 0.07b	0.74 ± 0.01a
Sb54	113.54 ± 5.85a	41.56 ± 1.24b	22.92 ± 1.96a	4.83 ± 0.26a	12.62 ± 0.23a	2.08 ± 0.12a	17.61 ± 0.39a	45.27 ± 0.55c	3.85 ± 0.20a	15.58 ± 0.38a	1.63 ± 0.07b	0.62 ± 0.02b
Sb55	116.76 ± 6.17a	44.03 ± 1.10a	24.20 ± 1.17a	4.52 ± 0.19a	14.01 ± 0.53a	2.35 ± 0.06a	19.37 ± 0.75a	42.95 ± 0.68c	4.37 ± 0.17a	15.61 ± 0.36a	1.76 ± 0.05b	0.63 ± 0.02b
Sb56	120.02 ± 7.33a	45.68 ± 2.04a	18.88 ± 1.02a	4.23 ± 0.20a	12.49 ± 0.38a	2.00 ± 0.16a	18.40 ± 0.55a	49.95 ± 0.48b	3.94 ± 0.14a	14.76 ± 0.43a	1.82 ± 0.06b	0.69 ± 0.05b
Sb57	117.10 ± 4.35a	46.72 ± 1.59a	19.25 ± 0.99a	4.64 ± 0.17a	11.93 ± 0.36a	1.46 ± 0.14b	15.32 ± 0.38b	47.06 ± 0.53b	4.08 ± 0.19a	15.28 ± 0.49a	1.81 ± 0.05b	0.71 ± 0.04a
Sb58	118.14 ± 4.95a	42.51 ± 1.95a	23.05 ± 1.56a	4.73 ± 0.10a	12.03 ± 0.49a	1.49 ± 0.14b	14.46 ± 0.36b	52.77 ± 0.73a	4.26 ± 0.19a	15.02 ± 0.33a	1.91 ± 0.09b	0.73 ± 0.02a
Sb59	124.07 ± 3.04a	45.20 ± 2.33a	19.68 ± 1.17a	4.54 ± 0.23a	12.68 ± 0.45a	1.66 ± 0.17b	18.18 ± 0.58a	50.90 ± 0.86a	3.93 ± 0.17a	14.92 ± 0.17a	1.82 ± 0.04b	0.77 ± 0.02a
Sb60	114.44 ± 6.16a	39.68 ± 1.77b	17.75 ± 0.63b	4.81 ± 0.26a	10.73 ± 0.23b	1.35 ± 0.12b	15.39 ± 0.60b	45.91 ± 0.67c	3.89 ± 0.20a	14.28 ± 0.48a	1.97 ± 0.05b	0.74 ± 0.02a
Sb61	124.15 ± 5.59a	43.20 ± 2.97a	21.97 ± 2.47a	4.63 ± 0.19a	12.67 ± 0.45a	2.02 ± 0.50a	16.66 ± 0.44a	49.10 ± 0.75b	4.04 ± 0.15a	14.62 ± 0.56a	1.94 ± 0.07b	0.70 ± 0.02a
Sb62	119.44 ± 5.72a	42.06 ± 0.99b	17.60 ± 1.04b	4.92 ± 0.21a	11.16 ± 0.24b	1.86 ± 0.14a	15.79 ± 0.35b	46.18 ± 0.56c	3.82 ± 0.21a	13.80 ± 0.43b	2.09 ± 0.05a	0.75 ± 0.02a
Sb63	126.24 ± 5.39a	42.57 ± 1.84a	17.01 ± 1.12b	4.50 ± 0.12a	10.96 ± 0.33b	1.50 ± 0.16b	14.92 ± 0.35b	51.21 ± 0.47a	4.15 ± 0.17a	14.55 ± 0.20a	2.22 ± 0.07a	0.72 ± 0.02a
Sb64	127.02 ± 3.33a	42.26 ± 2.91a	18.59 ± 0.86b	5.03 ± 0.19a	12.17 ± 0.35a	1.89 ± 0.18a	16.94 ± 0.28a	47.67 ± 0.37b	3.86 ± 0.15a	14.04 ± 0.37b	2.18 ± 0.04a	0.77 ± 0.03a
Sb66	114.21 ± 2.17a	41.75 ± 1.71b	15.98 ± 0.61b	4.91 ± 0.20a	11.23 ± 0.15b	1.24 ± 0.11b	16.47 ± 0.22a	22.00 ± 1.12f	4.14 ± 0.13a	14.19 ± 0.18a	2.00 ± 0.05b	0.68 ± 0.02b
Karahan	112.95 ± 2.36a	40.49 ± 1.55b	18.53 ± 1.03b	4.33 ± 0.19a	11.73 ± 0.31a	1.54 ± 0.15b	17.58 ± 0.39a	44.61 ± 0.78c	4.09 ± 0.10a	14.83 ± 0.43a	1.92 ± 0.10b	0.67 ± 0.02b
