# Supplementary material for: Multi-Year Characterisation of Drought Adaptation and Grain Quality in Synthetic Hexaploid Wheat Genotypes
Source: Plants (Basel). 2026 Oct 7;15(19):3054. doi: 10.3390/plants15193054 (PMC13666747; doi:10.3390/plants15193054)
Supplement: Supplementary file 1 [file plants-15-03054-s001.zip › Supplementary Table S2.pdf]

Table S2. Summary of selected synthetic wheat genotypes based on drought tolerance indices.

Genotype	MP	GMP	STI	Overall interpretation
Sb7	4.328	4.318	1.201	Good combination of yield potential and drought tolerance
Sb8	4.400	4.399	1.246	High productivity with favorable performance across contrasting environments
Sb9	4.339	4.317	1.200	High performance, particularly under water-limited conditions
Sb29	4.392	4.392	1.242	Balanced performance under contrasting water regimes
Sb30	4.328	4.326	1.206	High productivity index values
Sb40	4.367	4.362	1.225	High productivity with favorable stress response
