# Supplementary material for: Multi-Year Characterisation of Drought Adaptation and Grain Quality in Synthetic Hexaploid Wheat Genotypes
Source: Plants (Basel). 2026 Oct 7;15(19):3054. doi: 10.3390/plants15193054 (PMC13666747; doi:10.3390/plants15193054)
Supplement: Supplementary file 1 [file plants-15-03054-s001.zip › Supplementary Table S3.pdf]

Table S3. Meteorological data for the 2022–2025 Growing Season at the the Absheron Scientific Research Base Experimental Station of the Institute of Genetic Resources, Azerbaijan

Months	10-day intervals during the growing season	Air temperature, ^{0}C				Average relative humidity of the air, %	Maximum wind speed in m/s	Total precipitation (mm)		
		Long-term average temperature	Average monthly	Maximum temperature	Minimum			10-day intervals	Monthly	Average multi-year
1	2	3	4	5	6	7	8	9	10	11
2022-2023 growing season										
September	I	19,0	21,8	31,2	11,9	64	10	18,5	64,5	31,0
	II	17,2	15,2	24,4	9,7	76	9	42,9		
	III	15,2	16,3	25,1	6,9	74	10	3,1		
October	I	13,1	14,9	24,4	6,9	77	22	8,6	20,5	45,0
	II	11.1	11,3	20,7	2,2	81	12	11,9		
	III	9,4	15,2	27,4	7,4	67	19	--		
November	I	7,7	12,8	21,4	7,7	71	18	31,8	80,0	36,0
	II	5,8	10,7	24,0	4,4	71	24	38,0		
	III	4.5	8,4	19,1	0,2	71	25	10,2		
December	I	3,6	8,0	18,5	0,9	75	21	16,3	91,5	30,0
	II	1,6	3,2	16,2	-2,2	87	12	32,2		
	III	0,9	4,4	14,4	-1,8	70	21	43,0		
2023-2024 growing season										
January	I	0.2	5,1	15,0	-2,9	69	22	37,0	62,2	26,0
	II	-0.5	-0,1	12,7	-9,6	77	18	10,7		
	III	-0.4	-0,4	14,3	-6,6	84	12	14,5		
February	I	-0.3	4,7	16,9	-3,4	63	24	14,0	23.2	35,0
	II	-0.1	4,8	19,3	-3.5	71	13	3,2		
	III	0.8	-1,8	5,8	-11,3	84	9	6,0		
March	I	1.7	2,5	12,3	-5,9	88	18	12,4	49,7	42,0
	II	2.7	5,1	12,5	-0,3	86	18	26,0		
	III	4.8	7,4	18,2	-0,8	81	18	11,3		
April	I	7.1	10,9	18,5	3,9	72	12	20,0	21,1	47,0
	II	9.3	14,9	26,5	3,1	61	11	0,5		
	III	11.2	16,8	28,2	6,9	56	8	0,6		
May	I	13.1	14,7	25,4	5,1	72	10	36,8	89,2	47,0
	II	15.1	12,2	26,0	4,6	81	10	49,3		
	III	16.6	17,3	32,4	5,0	66	8	3,1		
June	I	18.1	22,9	31,7	14,3	60	8	39,0	145,2	40,0
	II	19.6	23,8	33,8	15,8	67	8	43,0		
	III	20.7	20,2	32,6	13,1	71	8	63,2		
July	I	21.9	27,9	35,6	14,4	51	8	20	29,8	14,0
	II	23.1	24,8	33,1	15,9	57	7	--		
	III	22.9	22,6	32,5	15,4	66	8	9,8		

August	I	22.7	25,4	35,4	17,2	56	8	0,3	14,8	13,0
	II	22.4	22,1	31,7	12,7	62	8	14,2		
	III	20.8	25,2	34,1	13,9	42	9	0,3		
Average		10,63	13,1					Total	691,7	406,0

2023-2024 growing season

Months	10-day intervals during the growing season	Air temperature, ^0C				Average relative humidity of the air, %	Maximum wind speed in m/s	Total precipitation (mm)		
		Long-term average temperature	Average monthly	Maximum temperature	Minimum			10-day intervals	Monthly	Average multi-year
1	2	3	4	5	6	7	8	9	10	11
2023 growing season										
September	I	19,0	21,2	37,4	12,0	48	9	0,5	6,0	31,0
	II	17,2	20,2	30,0	11,0	49	18	2,3		
	III	15,2	19,1	30,5	6,6	59	19	3,2		
October	I	13,1	17,9	27,3	10,0	62	9	--	21.0	45,0
	II	11.1	13,9	24,9	8,3	80	19	6,1		
	III	9,4	9,9	18,3	3,8	95	13	14,9		
November	I	7,7	7,2	16,2	1,4	80	13	20,1	81,4	36,0
	II	5,8	7,4	21,1	0,5	85	22	30,3		
	III	4.5	8,5	18,5	0.5	83	13	31,0		
December	I	3,6	-0,1	8,4	-3,8	94	9	10,0	11,0	30,0
	II	1,6	3,9	12,8	-0,7	90	12	1.0		
	III	0,9	2,3	7,2	-3,4	87	18	--		
2024 growing season										
January	I	0.2	1,8	14,4	-7,3	70	11	6,2	28,6	26,0
	II	-0.5	1,1	4,9	-7,2	93	9	20,9		
	III	-0.4	0,6	6,1	-6,9	87	9	1,5		
February	I	-0.3	0,3	5,9	-5,4	91	12	28,0	43,5	35,0
	II	-0.1	-0,3	14,8	-8,5	72	22	7,8		
	III	0.8	3,3	17,5	-5,3	48	25	7,7		
March	I	1.7	7,2	18,4	0.6	72	22	0,7	4.4	42,0
	II	2.7	10,7	21,7	3,1	67	22	1,5		
	III	4.8	10,6	22,5	0,3	63	19	2,2		
April	I	7.1	7,8	16,9	-0,6	82	11	19,7	35,9	47,0
	II	9.3	11,0	23,6	0,5	90	22	13,0		
	III	11.2	16,1	27,1	7,4	67	11	3,2		
May	I	13.1	13,0	23,1	6,4	77	10	60,7	68,3	47,0
	II	15.1	12,7	23,8	5,7	74	9	4,9		

	III	16.6	21,8	33,6	12,9	57	10	2,7		
June	I	18.1	20,4	32,0	11,7	64	10	2,4	4,1	40,0
	II	19.6	23,4	32,8	15,0	68	10	1,2		
	III	20.7	21,1	32,1	10,8	62	10	0,5		
July	I	21.9	24,2	36,8	13,0	66	10	10,3	31,0	14,0
	II	23.1	21,5	31,6	13,4	73	12	20,7		
	III	22.9	25,4	35,9	16,1	66	11	--		
August	I	22.7	24,8	36,1	19,0	61	11	--	21,1	13,0
	II	22.4	25,7	37,1	16,4	64	11	0,8		
	III	20.8	22.2	34,9	15,3	77	11	20,3		
Average		10,63	12,7					Total	356,3	406,0

2024-2025- growing season

Months	10-day intervals during the growing season	Air temperature, ^{0}C				Average relative humidity of the air, %	Maximum wind speed in m/s	Total precipitation (mm)		
		Long-term average temperature	Average monthly	Maximum temperature	Minimum			10-day intervals	Monthly	Average multi-year
1	2	3	4	5	6	7	8	9	10	11
2024- growing season										
January	I	0.2	3,1	13,9	-2,9	74	20	--	22.0	26,0
	II	-0.5	0,2	14,1	-7,4	80	22	21,3		
	III	-0.4	-0,4	9,4	-7,6	74	22	0,7		
February	I	-0.3	4,1	15,4	-4,9	62	22	2,4	14,7	35,0
	II	-0.1	2,1	13,4	-2,7	82	18	12,3		
	III	0.8	5,8	14,6	-1,2	78	22	--		
March	I	1.7	4,5	13,4	-3,6	75	30	5,8	41,0	42,0
	II	2.7	-0,7	-3.4	-3,6	88	11	32,4		
	III	4.8	3,1	18,8	-3,4	72	30	2,8		
April	I	7.1	12,4	28,0	2,2	56	22	1,2	1,2	47,0
	II	9.3	11,7	27,8	0,1	55	18	--		
	III	11.2	15,4	26,9	5,2	60	12	--		
May	I	13.1	12,9	22,4	7,0	72	13	52,6	97,3	47,0
	II	15.1	14,3	26,5	4,0	69	13	7,6		
	III	16.6	15,5	31,0	5,8	72	13	37,1		
June	I	18.1	22,0	34,0	10,0	48	13	19,0	20,5	40,0
	II	19.6	21,5	31,0	12,3	56	11	1,5		
	III	20.7	24,0	33,0	14,7	52	10	--		
July	I	21.9	21,7	33,3	10,6	43	11	5,4	25,6	14,0
	II	23.1	28,5	39,1	16,2	28	12	--		
	III	22.9	23,0	32,9	21,0	43	9	20,2		
August	I	22.7	24,2	34,2	16,8	69	10	5,6	5,6	13,0
	II	22.4	25,4	36,9	14,0	30	10	--		
	III	20.8	22,2	32,5	11,3	35	11	--		

Average	10,63	11,95		Total	288,1	406,0
