# Supplementary material for: Multi-Year Characterisation of Drought Adaptation and Grain Quality in Synthetic Hexaploid Wheat Genotypes
Source: Plants (Basel). 2026 Oct 7;15(19):3054. doi: 10.3390/plants15193054 (PMC13666747; doi:10.3390/plants15193054)
Supplement: Supplementary file 1 [file plants-15-03054-s001.zip › Supplementary Table S4.pdf]

Table S4. Irrigation schedule applied in the irrigated treatment during the winter wheat growing seasons

Irrigation event	Approximate timing	Growth stage of winter wheat	Purpose of irrigation	Applied water amount (m^3 ha^{-1})
1st irrigation	Early March	Spring regrowth and tillering	To stimulate vegetative growth after winter dormancy and support tiller development	500–600
2nd irrigation	Early April	Stem elongation	To support rapid biomass accumulation and spike development	500–600
3rd irrigation	Late April – Early May	Flag leaf emergence	To maintain photosynthetic activity and protect yield potential	500–600
4th irrigation	Mid-May	Flowering (anthesis)	To ensure adequate water availability during pollination and early grain formation	500–600
5th irrigation	Late May – Early June	Grain filling	To support kernel development and improve thousand-kernel weight and grain quality	400–500
