# Supplementary material for: Multi-Year Characterisation of Drought Adaptation and Grain Quality in Synthetic Hexaploid Wheat Genotypes
Source: Plants (Basel). 2026 Oct 7;15(19):3054. doi: 10.3390/plants15193054 (PMC13666747; doi:10.3390/plants15193054)
Supplement: Supplementary file 1 [file plants-15-03054-s001.zip › Supplementary Table S5.pdf]

Supplementary Table S5.

Table S5. Soil water content (θ) and field capacity (FC) across the growing season.

Soil Depth (cm)	FC (% Weight)	Dryθ—Early (%)	Seasonθ—Mid (%)	Seasonθ—Late Season (%)
0–30	42.5	42.3	37.1	30.1
30–60	40.0	40.2	35.0	28.5
60–90	38.5	38.7	33.9	27.0
